# Nurse prescribing of medicines in Western European and Anglo-Saxon countries: a systematic review of the literature

**DOI:** 10.1186/1472-6963-11-127

**Published:** 2011-05-27

**Authors:** Marieke Kroezen, Liset van Dijk, Peter P Groenewegen, Anneke L Francke

**Affiliations:** 1 NIVEL, Netherlands Institute for Health Services Research, PO Box 1568, 3500 BN Utrecht, The Netherlands; 2 Department of Sociology and Department of Human Geography, Utrecht University, Utrecht, The Netherlands; 3 Department of Public and Occupational Health, EMGO Institute for Health and Care Research (EMGO+) of VU University Medical Center, Amsterdam, The Netherlands

## Abstract

**Background:**

A growing number of countries are introducing some form of nurse prescribing. However, international reviews concerning nurse prescribing are scarce and lack a systematic and theoretical approach. The aim of this review was twofold: firstly, to gain insight into the scientific and professional literature describing the extent to and the ways in which nurse prescribing has been realised or is being introduced in Western European and Anglo-Saxon countries; secondly, to identify possible mechanisms underlying the introduction and organisation of nurse prescribing on the basis of Abbott's theory on the division of professional labor.

**Methods:**

A comprehensive search of six literature databases and seven websites was performed without any limitation as to date of publication, language or country. Additionally, experts in the field of nurse prescribing were consulted. A three stage inclusion process, consisting of initial sifting, more detailed selection and checking full-text publications, was performed independently by pairs of reviewers. Data were synthesized using narrative and tabular methods.

**Results:**

One hundred and twenty-four publications met the inclusion criteria. So far, seven Western European and Anglo-Saxon countries have implemented nurse prescribing of medicines, viz., Australia, Canada, Ireland, New Zealand, Sweden, the UK and the USA. The Netherlands and Spain are in the process of introducing nurse prescribing. A diversity of external and internal forces has led to the introduction of nurse prescribing internationally. The legal, educational and organizational conditions under which nurses prescribe medicines vary considerably between countries; from situations where nurses prescribe independently to situations in which prescribing by nurses is only allowed under strict conditions and supervision of physicians.

**Conclusions:**

Differences between countries are reflected in the jurisdictional settlements between the nursing and medical professions concerning prescribing. In some countries, nurses share (full) jurisdiction with the medical profession, whereas in other countries nurses prescribe in a subordinate position. In most countries the jurisdiction over prescribing remains predominantly with the medical profession. There seems to be a mechanism linking the jurisdictional settlements between professions with the forces that led to the introduction of nurse prescribing. Forces focussing on efficiency appear to lead to more extensive prescribing rights.

## Background

The number of countries where nurses are legally permitted to prescribe medication has grown considerably over the last two decades [1,2]. However, even though the term 'nurse prescribing' suffices as descriptor term, the actual practice it refers to varies considerably, both within countries and internationally [3]. Still, international comparisons with regard to nurse prescribing are scarce and those reviews that make an international comparison either focus on the effects of nurse prescribing [4], or lack a clear theoretical and systematic approach [5,6]. A comparative review of the extent of, and the ways in which nurse prescribing has been realised or is being initiated internationally, supported by a sound theoretical model, is lacking. The way in which prescribing by nurses is organized has far-reaching implications, both for the allocation of jurisdictional control over prescriptive authority and for the potential success of nurse prescribing in daily practice. Theoretical insights can help to shed light on these relationships. We therefore set out an international systematic review of publications dealing with the implementation process of nurse prescribing and current nurse prescribing practices within Western European and Anglo-Saxon countries. The theoretical framework used in the review is based on Andrew Abbott's theory on the division of expert labor in modern societies [7].

Traditionally, the task of prescribing medicines has been the domain of the medical profession [8,9], but the development of nurse prescribing represents an incursion on the medical profession's jurisdiction over prescribing. According to Abbott [7], jurisdiction - 'the link between a profession and its work' - forms the central phenomenon of professional life. Since one profession can pre-empt another's jurisdiction or control over a task, professions exist in an interdependent system with competing jurisdictional claims. These claims can be made in several arenas, i.e. professions can claim control over tasks in the legal arena, the workplace and in the arena of public opinion.

Abbott [7] extensively discusses the internal and external forces that shape professional competition over jurisdiction. Examples of external and internal forces that could possibly shape professional competition over prescribing rights are, respectively, striving for a more cost-effective healthcare system and a shortage of doctors within the healthcare workforce [10]. However, 'there are only so many full jurisdictions to go around' [7]. Consequently, most professional conflicts over jurisdiction result in so-called 'limited jurisdictional settlements', of which Abbott distinguishes five:

- *Subordination: *the second most desired outcome of a jurisdictional conflict, as the incumbent profession controls the division of labor in which one or more subordinate groups take their place.

- *Intellectual jurisdiction: *in which the incumbent profession controls the cognitive knowledge of an area but allows practice by other professions.

- *Division of labor: *in which the jurisdiction over a certain task is divided between professions into 'functionally interdependent but structurally equal parts'.

- *Advisory jurisdiction: *the weakest form of control, whereby a profession seeks 'a legitimate right to interpret, buffer or partially modify actions another takes within its own full jurisdiction'.

- *Client differentiation: *in which segments of a profession serve different client groups. This is considered a workplace settlement by Abbott.

**Figure 1:**
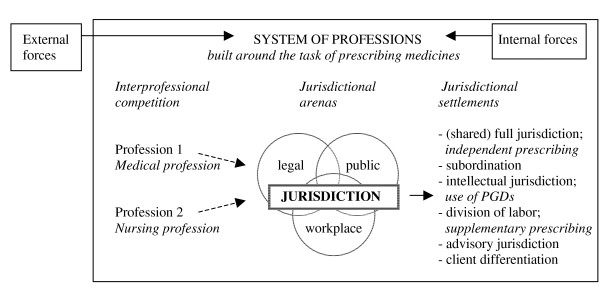
Graphic and partial depiction of Abbott's theory applied to the case of nurse prescribing.

Figure 1 shows a graphic and partial representation of Abbott's theory, applied to the case of nurse prescribing.

Although this article focuses on the introduction and realization of legal nurse prescribing, potential jurisdictional claims over prescribing held by one of the involved professions in other arenas were also included in our model, since they might influence claims made in the legal arena. For example in the United States of America, as Abbott [7] states, it is 'through public opinion that professions establish the power that enables them to achieve legal protection'. And as Sampson [11] states, a strong cohesive nursing community, grassroots legislative constituency and patient support are crucial in political battles over prescribing rights. We also applied Abbott's potential settlements of a jurisdictional conflict to the case of nurse prescribing (see Figure 1). For this purpose, the three general models of (nurse) prescribing usually distinguished in the literature were used as a point of departure:

### Independent prescribing

Legally permitted and qualified independent prescribers are responsible for the clinical assessment of a patient, the establishment of a diagnosis and decisions about the appropriateness of a medication, treatment or appliance, including the issuing of a prescription [12,13]. Prescribing usually takes place from a limited formulary - a list containing a limited and defined number of medicines that can be prescribed - or an open formulary. This type of prescribing is also referred to as initial, autonomous, substitutive and open prescribing [4,14]. Where nurses are able to independently prescribe medicines, with a fair range of prescribing freedom concerning medicine choice, we considered both the nursing and the medical profession to hold equal and full jurisdiction over prescribing, according to Abbott's classification (see Figure 1). It should be noted however that this is an exceptional case, as it is very rare for two groups to hold equal jurisdiction in a particular task area [7].

### Supplementary prescribing

Supplementary prescribing is defined as a voluntary partnership between an independent prescriber - a doctor or a dentist - and a supplementary prescriber - usually a nurse or a pharmacist. After the initial assessment and diagnosis of a patient's condition have been carried out by the independent prescriber, the supplementary prescriber may prescribe from an open or limited formulary and will collaborate or consult with the independent prescriber before issuing the prescription, even though direct supervision is not required [13-16]. Because of the clear delineation of areas of responsibility, we considered supplementary prescribing as a 'division of labor' in Abbott's terms (see Figure 1).

In the United Kingdom, an important additional feature of supplementary prescribing is formed by the collaboration between the independent and supplementary prescribers in drawing up a Clinical Management Plan which needs to be approved by the patient before implementation [15,16]. Supplementary prescribing is also known as dependent, collaborative, semi-autonomous or complementary prescribing [4,14].

### Patient group directions

Patient group directions (PGDs), formerly known as group protocols, refer to written instructions for the supply and administration of named medicines in an identified clinical situation [4,14,17,18]. Drawn up by a multidisciplinary team, they are specifically designed for a particular group of patients with a specific condition, thus excluding individualised prescriptions [19]. Group protocols should not be seen as independent prescribing, since nurses or other health care professionals are only allowed to supply and administer medications within the strict terms of a predetermined protocol, albeit using their own assessment of patient need [16,18]. Because PGDs are developed by a multidisciplinary team - usually consisting of doctors, pharmacists and nurses - we considered the 'intellectual jurisdiction' over the prescribing task to lie with the team, according to Abbott's classification, even though the nurse performs the actual task (see Figure 1).

Following Ryan, Cash and Hannis [20], 'time and dose prescribing', a fourth model sometimes distinguished in the literature, was not considered as a form of nurse prescribing in this review, as nurses are only allowed to alter the time and/or dosage of a particular medication. Furthermore, whilst the use of PGDs is not an actual form of prescribing, we nevertheless decided to include PGDs as a third model of prescribing in our study, considering their omnipresence in much of the nurse prescribing literature. Moreover, when using PGDs nurses do make a decision that refers to the medication itself, whereas with time and dose prescribing the decision to start with a particular medication has already been taken.

This article reports on the findings of a systematic review of the scientific and professional literature concerning nurse prescribing. The review is the first phase in a larger research project focussing on nurse prescribing and has a twofold aim. Firstly, to gain insight into the scientific and professional literature describing the extent to and the ways in which nurse prescribing has been realised or is being initiated in Western European and Anglo-Saxon countries. Secondly, to propose possible mechanisms underlying the organisation of nurse prescribing internationally, and relate these to Abbott's theory on the division of expert labor [7].

The following questions were addressed:

1. To what extent has nurse prescribing of medicines been initiated or already realised in Western European and Anglo-Saxon countries?

2. As a result of which external and internal forces has nurse prescribing been initiated or already realised in Western European and Anglo-Saxon countries?

3. Under which legal, educational and organizational conditions are nurses allowed to prescribe medicines within Western European and Anglo-Saxon countries?

4. Which jurisdictional settlements can be discerned between the medical and nursing professions concerning the task of prescribing medicines?

5. Which mechanism, if any, can be discerned between the forces that lead to the introduction of nurse prescribing and the resulting jurisdictional settlements between the medical and nursing professions?

## Methods

### Search strategy

The following six electronic databases were searched without any limitation as to date of publication or language: PubMed, Embase, CINAHL, Web of Science, EBSCO Academic Search Elite and the NIVEL-catalogue. Searches were highly sensitive, using the following search strategy for PubMed: ("Nurse prescribing") or (Nurs* [tiab] AND Prescri* [tiab]) or (Nurses [MeSH] AND "drug prescriptions" [MeSH]) or (Nurses [MeSH] AND formulary [tiab]). Suitable search strategies were developed for the other databases, using adaptations of the PubMed search. All detailed search strategies can be found in additional file 1 'Search strategies'.

In addition to the electronic databases, the following relevant websites were searched: the website of the Virginia Henderson International Nursing Library http://www.nursinglibrary.org, the website of the World Health Organization http://www.who.int, websites for health professionals http://www.nurse-prescriber.co.uk, http://www.nursingtimes.net, http://www.escriber.com, http://www.internurse.com and Google Scholar http://scholar.google.com. Since most of these websites lacked advanced search facilities, the following keywords were used to search for relevant publications: "nurse prescribing", "independent (nurse) prescribing", "autonomous prescribing" "supplementary (nurse) prescribing", "dependent (nurse) prescribing", "collaborative prescribing", "group protocols" "patient group directions", "time and dose prescribing", "nurse formulary" and combinations of these keywords. All detailed search strategies can be found in additional file 1 'Search strategies'. Additionally, we consulted experts in the field to identify any studies that might have been missed.

The hits of all searches were entered into Reference Manager©; duplicates were sifted out in this program, and the inclusion process was executed thereafter.

### Study selection

Publications from 2005 onwards had to fulfil all of the following criteria in order to be included:

1) The publication concerns a situation in which legal nurse prescribing of medicines is being initiated or has already been realised. We considered legal nurse prescribing as 'being initiated' if at least a change in the law, or new legislation enabling nurses to prescribe medicines was in preparation, either at national, provincial or state level.

2) The publication addresses legal nurse prescribing of medicines within the geographical context of at least one Western European or Anglo-Saxon country. Since the definition of Western Europe is complex and carries economic and cultural connotations, we adopted the definition of the renowned National Geographic Society.

3) The publication specifies either the external or internal forces under which legal nurse prescribing has been initiated or realised, or the legal, educational or organizational conditions under which nurses are allowed to prescribe medicines.

4) The group of professionals with prescribing rights discussed in the publication includes registered nurses (but not Physician Assistants).

5) The publication is a professionally or scholarly 'sound' publication, i.e. a scientifically peer reviewed publication or a publication by a government body or professional association.

Because we aimed to describe nurse prescribing as it is currently being initiated or has been realised in Western European and Anglo-Saxon countries, publications from 2005 and later had to meet all the inclusion criteria. However, in view of our comparative theoretical framework, we were also interested in the external and internal forces that led to the introduction of nurse prescribing and which influence the system of professions and the division of jurisdictions between professions. As these forces are mainly found in publications dating from the period of introduction, and nurse prescribing has been established in some countries for years, publications prior to 2005 were also included in the review. However, as our review is only concerned with contemporary nurse prescribing practices, publications prior to 2005 did not have to fulfil the second part of inclusion criterion 3, i.e. they did not have to address the conditions under which nurses are allowed to prescribe medicines.

Publications were excluded if:

1) They focussed on legal nurse prescribing in countries other than Western European and Anglo-Saxon countries.

2) They exclusively related to legal nurse prescribing of appliances and dressings and made no reference to legal nurse prescribing of medicines.

3) They only concerned nurse prescribing by specified group protocols that severely limit the prescribing rights of nurses, more specifically group protocols for (emergency) contraception, child and travel vaccinations and annual influenza vaccinations.

4) They merely related to time and dose prescribing.

5) They focussed solely on illegal rather than legal nurse prescribing of medicines.

6) They only discussed the prescribing rights of midwives and/or nurses holding midwifery credentials - the latter only if their prescribing rights were based on their midwifery credentials or if uncertainty existed about the underpinning of their prescribing rights.

In some cases the boundary between nurses and midwives proved blurred, for example in the case of the American certified nurse-midwife, who is an advanced practice nurse with specialized education and training in both nursing and midwifery. We adopted a consistent approach to this issue and excluded all midwives from the review. Specialised nurses working in an obstetrics department without holding a midwifery certification were included.

A three-stage inclusion process was applied. All references found in the literature search of databases and websites were initially studied independently by title and abstract by pairs of reviewers (MK, ALF and LvD) and included in the study if they met the above mentioned criteria. All references deemed eligible for inclusion by at least one reviewer proceeded to the next selection round.

In the second stage, pairs of reviewers (MK, ALF and LvD) independently examined the remaining references once more by title and abstract. References from 2005 onwards that - on closer scrutiny - did not meet all inclusion criteria were excluded. All references prior to 2005 that did not explain the external or internal forces under which nurse prescribing was initiated or realised were likewise excluded. Again, all references deemed eligible for inclusion by at least one reviewer were included. However, because of the abundance of UK-based references selected in the first two stages, and the large number of internal and external forces mentioned in these references, the first author, after discussion with the other two reviewers, excluded all UK-based references prior to 2005 from the review before turning to the final selection round.

In the final stage, the full text of all remaining publications was obtained. Pairs of reviewers (MK, ALF and LvD) independently studied each publication in order to determine whether it fulfilled the inclusion criteria, and disagreements were resolved by discussion.

Where several publications were based on the same study, containing identical information, the first author only selected the most recent as well as the most elaborative publication for final inclusion in the review.

### Additional step during study selection

During the study selection process, the first reviewer drew up a list containing all Western European and Anglo-Saxon countries referred to in the titles and abstracts of the initial search results as having initiated or realised nurse prescribing. It was assumed that countries missing on the resulting list had not initiated or realised nurse prescribing. To make sure that this division into 'prescribing' and 'non-prescribing' countries corresponded with the current state of affairs across countries, we verified our findings with representatives of leading national nurses and medical associations and government representatives.

### Data synthesis and analysis

The first author (MK) extracted data from the included publications onto digital structured data-extraction forms, and two other authors (ALF and LvD) checked the extracted data. Disagreements were resolved by discussion between the review authors. Data were extracted on country, external and internal forces that led to the introduction of nurse prescribing; the educational and organizational criteria that must be fulfilled in order for nurses to prescribe medicines; the legal conditions in place; the financial issues with regard to nurse prescribing and; where appropriate, the models of nurse prescribing being used.

We used Abbott's theory on the division of labor as a point of departure to organize and summarize the data. Abbott pays considerable attention to the internal and external forces that shape professional competition over jurisdiction - in this case the jurisdiction over prescriptive authority. Moreover, he proposes a number of 'jurisdictional settlements' that are easily compatible with the three general models of nurse prescribing usually distinguished in the literature. These models mainly focus on the legal conditions in place. As educational and organizational conditions further determine the organization of nurse prescribing and hence the outcomes of jurisdictional conflicts, data were eventually organized under the following broad themes: forces related to the introduction of nurse prescribing; legal conditions under which nurse prescribing of medicines will be or has been realised; educational conditions under which nurse prescribing of medicines will be or has been realised; and the organizational conditions under which nurse prescribing of medicines will be or has been realised.

## Results

### Search and inclusion results

**Figure 2:**
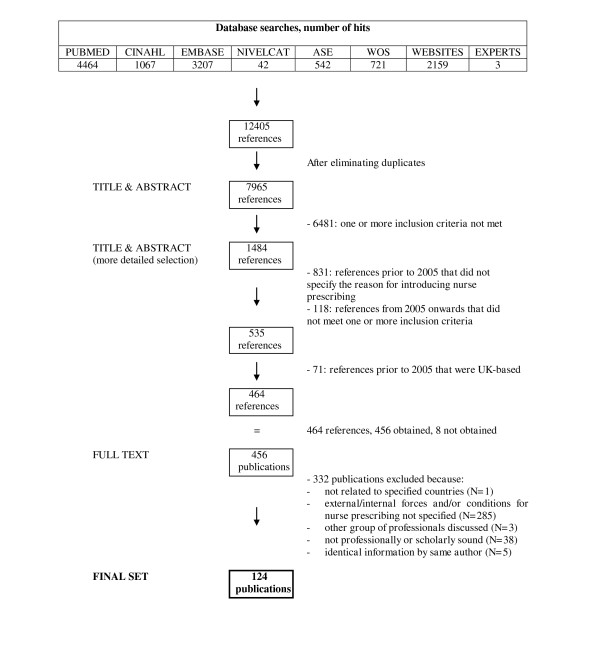
Flow diagram of study selection process.

After duplicates had been removed, the searches resulted in an initial set of 7965 references of potential interest. Following a first sifting based on title and abstract, 1484 references were selected for more detailed scrutiny by title and abstract. The resulting set of 464 articles was ordered in full text. After application of the inclusion criteria, 167 studies were deemed eligible for inclusion, of which 5 publications contained duplicate information by the same author and 38 publications did not live up to our 'soundness' criteria. Finally, 124 publications were selected for the next stage of the review, for data-extraction and analysis. Figure 2 shows the flow diagram of the inclusion process.

### Characteristics of the final 124 publications

#### Countries of interest

Additional file 2 'Characteristics of included publications' provides a descriptive overview of all included publications. The majority of included publications focussed on one country (N = 99) [21-119]. Of these, seventy-five publications were based in the United Kingdom, ten in the United States of America, five in New Zealand, four in the Netherlands, two in Ireland and the rest in Australia, Canada or Sweden. Twenty-three publications made reference to multiple countries, almost always including the UK and the USA [120-142]. Just two international comparative nurse prescribing publications were included in the review, covering 10 and 12 countries respectively [5,6].

As said before, it was assumed that countries not mentioned in the titles and/or abstracts of the search results had not initiated or realised nurse prescribing. We checked our findings regarding 'nurse prescribing countries' with relevant stakeholders across Western European and Anglo-Saxon countries (see additional file 3: Results of verification literature search with relevant stakeholders in Western European and Anglo-Saxon countries). This proved fruitful, as we were informed that an implementation process for nurse prescribing is currently being rolled out in Finland. However, since no literature on Finland was identified through our search strategy, Finland will not feature in our results section. From all other Western European and Anglo-Saxon countries that were not identified with our literature search, we received confirmation that nurses are indeed not allowed to prescribe medicines and no implementation process is being initiated.

### Date and type of publications

Most publications were published in 2008 and 2009. The oldest publication included in the review dates from 1982 and the most recent ones from 2010. Publications were derived from a variety of sources, including fifty-five journals and magazines, four books and three reports.

### Main focus of publications

There was much diversity as to the main focus of the included publications. Nevertheless, a number of recurring themes could be discerned, such as the views of nurses, doctors and other parties involved concerning nurse prescribing [45,47,51,53,81,94,108,109,115,121,139], prescribing behaviours of nurses [36,43,124,126,128,129,131,137], and nurse prescribing in relation to specific diseases [38,44,84,89,91-93,101,108,116,125] - most notably concerning nurse prescribing in mental health care [21,32,39,67,76,83,100,102,104-106,132,135]. Also, a number of publications focussed on the history and evolution of (nurse) prescribing of medicines, but these remained relatively limited [5,6,46,48,49,75,90,96,103,142].

### Nurse prescribing themes discussed

Four broad themes were considered to be relevant for the organization of nurse prescribing internationally and the outcomes of jurisdictional competition over the prescription of medicines. All publications were labelled with appropriate themes (see additional file 2: Characteristics of included publications). Table 1 provides an overview of publications per theme. The content of these themes will be discussed later. Additional file 4 provides a descriptive overview of nurse prescribing across Western European and Anglo-Saxon countries at national level.

**Table 1:** Identified themes of nurse prescribing

Nurse prescribing theme	Studied by
Internal and external forces related to the introduction of nurse prescribing	[5,6,21-42,44-47,51,52,54-56,58-69,73-76,79-90,92-94,96,97,100,102-104,106-112,114-118,120-125,127,128,130-138,140-142]

Legal conditions under which nurse prescribing of medicines will be or has been realised	[5,6,21,23-29,31-35,37-40,42-53,56-58,60,61,63-66,68-73,76,78,82-85,89,92,94,95,97,98,100-105,108-110,112-114,116-121,123-129,131-139,141,142]

Educational conditions under which nurse prescribing of medicines will be or has been realised	[5,6,21,22,24-29,32-34,37-40,42,44,46-53,55-57,60,63-67,69,71,72,76-79,82,84,85,88-95,97,99-101,104,105,107-110,112-114,116-118,122-128,131,132,134-142]

Organizational conditions under which nurse prescribing of medicines will be or has been realised	[5,6,22,23,27,29,40,42,46,47,50,55,64,65,69,71,72,77,79,85,89,91,92,95,99,116,125,128,129,135,137,142]

### Initiation and realization of nurse prescribing

#### Year of introduction

It is notable that nurse prescribing was introduced at very different points in time in the seven Western European and Anglo-Saxon countries that have so far realised nurse prescribing, viz. Australia, Canada, Ireland, New Zealand, Sweden, the United Kingdom and the United States of America. While nurse prescribing has been in place in the USA since the 1960s [5,6,30,107,115,123,128,134,135,138], it is a relatively new phenomenon in most other countries. Table 2 presents an overview of the (expected) year of introduction of nurse prescribing in Western European and Anglo-Saxon countries. While community nurses were the first group of nurses to start prescribing in the UK in 1998, one should note that in the years thereafter two other models of nurse prescribing were introduced there: in 2002 the form now known as 'independent prescribing' was implemented [5,6,24,32,48,49,56,68,72,76,84,87,100,101,121,124,136], followed by 'supplementary prescribing' in 2003 [5,6,24,26,27,32,35,36,40,42,46,47,49,52,56,62,67-69,72,76,78,81,83,91-94,101,104,121,123,124,136,138]. Currently, nurses in the Netherlands are awaiting for the final amendments to legislation to enable them to start prescribing [5,58,117,118], and in Spain the legal regulation of nurse prescribing is in the procedural phase [5].

**Table 2:** Year of introduction of nurse prescribing

Year of introduction	Country
1960s	United States of America [5,6,30,107,115,123,128,134,135,138]

Early 1990s	Canada [6]

1994	Sweden [5,6,85,112,125,127,136]

1998	United Kingdom [5,6,25,27,40,42,46,48,56,57,62,67-69,72,76,78,85,98,100,101,104,114,116,122,135]

2000	Australia [5,129]

2001	New Zealand [5,6,122,138]

2007	Ireland [5,55,115,139]

Expected in the near future	The Netherlands [5,117,118]

Expected in the future	Spain [5]

#### Forces related to the introduction of nurse prescribing

External and internal forces which led to the introduction of nurse prescribing were mentioned in hundred and two of the hundred and twenty-four publications included. In the Netherlands, the aim of task reallocation in the health care sector and more particularly the undesirable situation in which nurses prescribe medicines on an illegal basis, have been the main driving force behind the introduction of nurse prescribing [117,118]. The objective of creating quicker and more efficient patient access to medicines has also been highly influential in the introduction process of nurse prescribing internationally, especially within the UK and Ireland [21,25,26,29,31,32,34-37,39,42,46,47,51,52,55,58,61,62,64,67,69,76,82-84,87,89,90,94,100,102,104,106,112,114][116,120,128,132,135-137,140]. Another important force in this process has been the aim to make better use of nurses' skills and knowledge, and to improve the use of both health professionals' and patients' time [5,6,23,26,30,34,37,39,41,42,44,46,47,51,52,55,56,60,64,66-69,73-76,79,84,86,87,89,90,92,93,100,102,103,106,109][115-118,124,125,130,132-134,137]. Whereas these seem to have been the main drivers behind the introduction of nurse prescribing in the UK and Ireland, forces originating from within the health professions appear to have prevailed in other countries. In Australia, Canada, New Zealand, Sweden and the USA nurses were granted prescribing rights in order to reduce the workload of doctors and physicians, address the shortage of physicians - partly resulting from the growing specialisation of health professionals - and meet the medication needs of patients in remote areas who were often suffering as a result of a shortage of physicians [5,6,22,28,30,33,34,41,51,59,61,85,85,86,107,120-122,124,125,127,128,131,135,138]. Moreover, prescriptive authority for nurses in Canada, New Zealand and the USA followed the development of advanced practice nurse (APN) roles [5,61,74,124], which clearly connects their prescribing privileges with internal developments within the nursing profession.

#### Legal conditions regarding nurse prescribing

All Western-European and Anglo-Saxon countries that have realised or initiated nurse prescribing have imposed legal restrictions on which categories of nurses can prescribe medicines, what, how much and to whom they can prescribe, and whether they are allowed to do so on an independent basis or under the supervision of a physician. In most countries, these issues are regulated at national level, but in some, such as Australia, Canada and the USA, prescriptive authority is regulated at federal, state or regional level [5,6,28,85,125,129,131].

Table 3 offers an overview of prescriptive authority for nurses across Western European and Anglo-Saxon countries. Independent prescribing rights were granted to nurses across all countries that have introduced nurse prescribing or are set to do so in the (near) future. Some countries introduced other models of nurse prescribing as well, such as supplementary or collaborative prescribing - prescribing in partnership with a physician - or the use of Patient Group Directions (PGDs) or medical directives by nurses to supply and administer medicines to patients [6,24,27,39,40,61,64,71,72,78,105,128]. For example, in over half of the US states nurses have full independent prescriptive authority, whereas in other states mandatory collaboration with and/or supervision by a physician is required [5,6,28,54,59,75,96,124,135,137]. Likewise in the Netherlands in the future, Nurse Specialists will be allowed to prescribe on an independent basis, although this authority will be limited to a maximum 'experimental period' of five years [117], while specific categories of specialist nurses will prescribe through a model resembling supplementary prescribing [118].

**Table 3:** Prescriptive authority for nurses in Western European and Anglo-Saxon countries

	Prescriptive authority		
**Country**	**Independent**	**Collaborative/** **supplementary**	**Use of PGDs/** **medical directives**

Australia	√		√

Canada	√		√

Ireland	√		

Netherlands*	√	√	

New Zealand	√		

Spain*	√	√	

Sweden	√		

United Kingdom	√	√	√

United States of America	√	√	

* Intended form(s) of prescriptive authority, nurse prescribing is not yet legal (see table 2)

Even though nurses in all countries are (or will be) allowed to prescribe medicines on an independent basis, their scope of practice or freedom to act varies considerably, depending on whether or not protocols and/or formularies are in place and if so, how restrictive these are. In Ireland nurse prescribers may independently prescribe from an open formulary specific to their field of clinical practice [5,139] whereas in the UK independent prescribers can prescribe from the entire British National Formulary (BNF), including unlicensed medicines and some controlled drugs [5,24,26,35,44-46,48,49,51-53,63,65,68,69,76,78,83,84,92,94,100-103,105,108,109,113,126,128,136,137,139]. Supplementary prescribers in the UK can in addition prescribe all controlled drugs, provided they are listed in a clinical management plan agreed by the independent prescriber, nurse and patient [5,21,24,26,27,35,38,40,43-45,48-53,63,76,78,82,95,100,101,108,114,123-126]. Community practitioner nurse prescribers in the UK however, have their own more limited formulary to prescribe from [5,27] and in South Australia, every nurse practitioner has their own individual formulary of medicines from which to prescribe [129]. Most Australian states however, just as a number of American states, Canadian provinces and Sweden, have general limited formularies for nurse prescribers in place [5,6,28,85,90,112,125-127,136]. Other commonly used means to restrict nurses independent prescriptive authority are protocols. The Australian states of New South Wales and Queensland, a number of American states, Canadian provinces and the Netherlands all (will) use protocols in enabling nurse prescribing [28,58,61,118,119,133].

When it comes to legal restrictions regarding patients and/or medical conditions for which nurses are allowed to prescribe medicines, the UK has granted nurses the most extensive prescription privileges. Community practitioner nurse prescribers can prescribe for a number of common conditions, but both independent and supplementary nurse prescribers can prescribe for any medical condition or patient group within their clinical competence [5,25,27,35,40,50,52,63,68,69,71,78,84,89,92,94,95,100-102,105,109]. A PGD can in principle also be drawn up for any medical condition, but should be reserved for those situations where it offers 'an advantage for the patient without compromising patient safety' [40,72]. In most other countries however, restrictions apply. In Sweden, only district nurses and nurses working in elderly care may prescribe for 60 conditions [5,6,85,125,127,136] and in Ontario (Canada) nurses can only prescribe in primary care, long-term care and outpatient clinics [61]. In New Zealand, prescriptive authority was for a long time granted only to nurses working in specific areas of care [90,125,138,139] but this recently appears to have been expanded to include the whole NP scope of practice [5].

The formal responsibilities that nurse prescribers carry are clearly defined in most Western European and Anglo-Saxon countries. For example, in the Canadian province of British Columbia, registered nurses who initiate medicines are 'fully responsible and accountable' for their prescription [61], and in Massachusetts (USA) nurses likewise assume responsibility for prescribing [134]. As the prescription of medicines forms just one element in the medical care of a patient, formal responsibilities are also established for the related tasks in the treatment process, viz. accountability and responsibility for the clinical assessment of a patient and the establishment of a diagnosis. In Australia, for example, nursing curricula focus on 'taking full responsibility for patient's treatment' [5]. In the UK, responsibility for the various aspects of the treatment process differs between the three categories of nurse prescribers. Independent nurse prescribers and qualified community nurse prescribers are responsible for the clinical assessment and diagnosis of a patient and for decisions about the clinical management required, including prescribing [27,40,47,48,51,56,69,72,78,83,84,89,92,100,102,108-110,114,124-126,136-138]. Supplementary prescribers, however, are only responsible for the continuing care of a patient, including prescribing, whilst the collaborating independent prescriber shares the responsibility for prescribing and holds full responsibility for the assessment and diagnosis of a patient [25,40,47-49,51,56,63,69,72,78,84,100,103,123,124,135]. In the Netherlands likewise, specialist nurses are only allowed to prescribe medicines after a diagnosis has been made by a doctor [118].

#### Educational conditions regarding nurse prescribing

In all Western European and Anglo-Saxon countries that have realised legal nurse prescribing, nurses are required to successfully complete a prescribing course before they are allowed to start prescribing [55,88,107,112,114,122,123,131,134,137-139,141,142]. However, no specific training is required for UK nurses using PGDs, although most individual Trusts provide some in-house training [24,39,40,105].

Regarding the place that nurse prescribing training occupies within the various national education systems and the level at which it is provided, there are differences between countries. Education programmes for nurse prescribing in Ireland as well as independent and supplementary prescribing courses in the UK, which are combined into a 'dual qualification' [5,32,33,44,51,53,64,72,93,110,116,122-126], are offered on a stand-alone basis, i.e. they are not part of a regular nursing curriculum. However, training to prescribe from the British Nurse Prescribers Formulary for Community Practitioners is incorporated into Specialist Practitioner Programmes [5,6,22,27,40,49,107,138] and in Sweden prescribing training is part of the Primary Health Care Specialist Nursing programme, undertaken by all district nurses [5]. In the Netherlands, it is anticipated that independent prescribing for Nurse Specialists will become an obligatory component of the Masters programme of Advanced Nursing Practice [5,117], just as in New Zealand where preparation courses for nurse prescribing are offered within a Masters programme for advanced nursing practice or as a stand-alone Post Graduate Diploma (Prescribing) for nurses who already completed a Masters [5,6,22,27,40,49,107,138].

There are also differences between countries regarding the educational level of nurse prescribing training. Where most countries, including Australia, Canada, New Zealand, the Netherlands and the USA require nurses to complete a master level degree before they are allowed to prescribe independently, the Irish nurse prescribing training is awarded at level 8 in the Irish education system - which is comparable to Honours Bachelor Degree level - and in the UK prescribing courses are taught at undergraduate level 3 (degree level) [5,6,24,27-29,37,48,50,52,53,56,60,63-65,69,72,76,77,79,84,88,89,91,95,99,107-109,116,120,122-125,131][134,137,138,140-142]. This is remarkable when we recall that nurses in Ireland and especially nurses in the UK have very broad independent prescribing rights. In the Netherlands, specialist nurses who will prescribe through a model resembling supplementary prescribing will be trained at Bachelor degree level [118].

Criteria to enter prescribing courses are relatively similar across countries. One of the most important requirements for nurses internationally to enter prescribing programmes is sufficient clinical experience. However, the minimum number of years of clinical experience required varies. In Ireland and the UK, three years of clinical experience are required [5,29,42,53,71,84,108,109,124,126,137,140], whereas in New Zealand, nurses must have at least four years of clinical experience in their speciality area [107,122]. In Australia as of January 2010, nurses must have five years of clinical experience in their own field of practice, before they are eligible for endorsements as a nurse practitioner and hence for prescribing medicines [5]. Thus, it seems that the UK and Ireland have lower educational- and clinical experience requirements in place for nurse prescribing than other Western European and Anglo-Saxon countries.

Another important requirement that often needs to be fulfilled, for example in Australia [131], New Zealand [107] and the UK [37,38,42,53,65,84,95,99,137], is the ability of nurses to demonstrate clinical assessment and clinical decision-making skills. In the UK, additional prerequisites for potential nurse prescribers include nurses' ability to arrange for a Designated Medical Practitioner (DMP) who will supervise them during their practice period and they must occupy a post in which nurse prescribing will enhance patient care [5,26,40,50,52,53,77,95,97,108,109,125].

The content of training programmes for nurse prescribing seems to be fairly similar across countries. Swedish nurses attend lectures on pharmacology, pharmacovigilance (PV/PVG) and adverse drug reaction (ADR) reporting [5]. In Australia [5], Ireland [5], New Zealand [5,122,138] and the UK, pharmacology likewise constitutes an important topic in the prescribing training, just as the legal and ethical aspects of prescribing and clinical decision making [5,48-50,52,110,114,122,124,125,132,136]. In the literature, assessments performed during or at the end of the prescribing course were only specified for the British situation and could therefore not be compared across countries. In the UK these include the completion of a portfolio and an assessment of nurses' calculation skills, on which a 100% score must be attained for independent and supplementary prescribing [5,21,29,38,40,50,52,89,100,113,116,122,127].

#### Organizational conditions regarding nurse prescribing

The organizational conditions under which nurses are allowed to prescribe medicines in Western European and Anglo-Saxon countries are much less discussed in the literature than educational and legal conditions for nurse prescribing. It is nonetheless clear that most countries operate some sort of mandatory registration system in which nurse prescribers have to be registered before they are allowed to prescribe. In Australia, nurses have to submit a formulary of all the medicines they may prescribe to their respective Nursing Boards as part of their endorsement process [5,88]; in the Netherlands prescribing nurses must be registered in the 'BIG' registration system kept by the Ministry of Health [117,118]; and in Ireland [5,115,139], New Zealand [5,6,79], the UK [5,22,23,29,38,42,71,77,85,91,92,95,116,142] and the USA [134] nurse prescribers must register their qualification with their respective national regulatory nursing bodies.

In the UK, the Nursing and Midwifery Council (NMC) together with the National Prescribing Centre (NPC), have defined the 'standards of proficiency that underpin principles of prescribing practice' [27,87,137], and several UK-based publications refer to nurses' responsibility to maintain and update their prescribing knowledge, known as continuing professional development [5,32,33,35,40,50,60,62,89,136]. These topics nevertheless draw little attention in the literature and are virtually absent in publications relating to the other Western European and Anglo-Saxon countries that have realised nurse prescribing, with the exception of Ireland and New Zealand where continuing education and development are also being stressed [5].

The financial aspects of nurse prescribing were touched upon in a mere nine publications. In the UK, funding to undertake nurse prescribing training is made available from central government through local level organizations, such as workforce development confederations, strategic health authorities and local NHS Trusts [40,42,46,47,65,69,72]. However, medical supervisors of nurses during their practical training period in the prescribing course are generally not financially rewarded for their support [40,99]. Moreover, in the UK, access to a prescribing budget needs to be created for nurse prescribers before they can perform their role [40,85]. Another important point that has scarcely been touched upon in the literature is the reimbursement of prescriptions written by nurses. In New Zealand, if a nurse practitioner prescribes a medicine, the costs to the patient are the same as if a doctor prescribes [129]. However, in several states of the USA, the social welfare program Medicaid does not reimburse prescriptions written by nurses [135].

## Discussion

Nurse prescribing of medicines is a major area of interest in the scientific as well as professional literature, as shown by the high number of identified publications. This review provides insight into the diversity of external and internal forces which led to the introduction of nurse prescribing in the nine identified Western European and Anglo-Saxon countries, while shedding light on the variety of legal, educational and organizational conditions in place. Moreover, by applying Abbott's theory on the division of labor in modern societies, a variety of jurisdictional settlements between the nursing and medical professions concerning the task of prescribing were discerned.

### Models of nurse prescribing and jurisdictional settlements

In the introduction to this article we briefly discussed the three general models of (nurse) prescribing usually distinguished in the literature, viz. independent prescribing, supplementary prescribing and the use of patient group directions (PGDs). However, these models appear to be largely based on the situation in the UK and may be less applicable to nurses' prescriptive authority in other Western European and Anglo-Saxon countries. For example, we found that nurses in Sweden and Ontario are only allowed to independently prescribe for a limited number of medical conditions. Hence, their prescribing practices do not fit with the common definition of 'independent prescribing' in which nurses enjoy unrestricted independent prescribing freedom with regard to medical conditions.

However, broadly speaking, all nine Western European and Anglo-Saxon countries identified in this review grant some form of independent prescribing authority to nurses, albeit with varying levels of autonomy. But where we considered 'independent prescribing' in the introduction as a situation in which both the nursing and medical professions hold equal and full jurisdiction over prescribing, according to Abbott's classification, this does not hold for all countries. Only in Ireland and the UK, where nurses' scope of prescribing practice is fairly extensive, did the level of autonomy prove sufficient to consider both the nursing and medical professions to hold equal and full jurisdiction over prescribing. All the other countries imposed such stringent restrictions on nurses' independent prescriptive authority via protocols and/or limited formularies of medicines, that the medical profession still has exclusive full jurisdiction over the prescribing task. Since nurses are often only allowed to prescribe relatively harmless medication in these countries, the medical profession has delegated to them the 'routine' part of prescribing and remains in control over the complex and professionally more important part. Hence, nurses prescribe on the basis of a subordinate jurisdiction.

Moreover, some countries such as Sweden not only place restrictions on the medicines that nurses are allowed to prescribe, but also on the type of patients for whom nurses may prescribe. Because of the inclusion of elements of client differentiation, we consider this an even more restrictive form of subordinate jurisdiction, thereby disputing Abbott's assumption that client differentiation is only a workplace settlement.

It is possible that these subordinate settlements of nurse prescribing constitute phases in a process towards shared full jurisdiction for the nursing profession. After all, the road towards extensive prescribing rights for nurses in the UK was also a gradual process, and we note that in New Zealand prescriptive authority was recently expanded to include the whole NP scope of practice [5]. Nonetheless, movements in countries other than the UK are generally slow. In some countries, hardly any developments have been made since the initial introduction of nurse prescribing, even though nurse prescribing was sometimes introduced at a (much) earlier point in time, such as in Sweden and the USA.

Whereas all nine Western European and Anglo-Saxon countries identified in this review have granted independent prescribing authority to nurses, some of them introduced other models of nurse prescribing as well, resulting in a variety of jurisdictional settlements. The requirements of several American states regarding physician involvement in nurse prescribing creates a model of prescriptive authority comparable to supplementary prescribing in the UK. In the Netherlands specific categories of specialist nurses will in the future also prescribe through a model resembling supplementary prescribing. Because of the clear distinction between areas of responsibility, we consider both supplementary prescribing and collaborative/supervised prescribing as forms of prescribing within a 'full division of labor', in Abbott's terms. PGDs and medical directives, on the contrary, are developed by a multidisciplinary team and a physician respectively, while the nurse is the one who uses them in daily practice. Hence, the 'intellectual jurisdiction' over the prescribing task lies with the developers.

Applying Abbott's classification system of jurisdictional settlements to the prescribing scope of nurses in Western European and Anglo-Saxon countries, it is clear that the jurisdiction over the prescribing task in most countries, apart from the UK and Ireland, remains predominantly with the medical profession.

### Mechanisms

In view of the extensive prescribing privileges that nurses in Ireland and especially the UK enjoy, it is remarkable that requirements concerning number of years of clinical experience and educational level in these two countries proved less stringent than in other Western-European and Anglo-Saxon countries. Nurse prescribing training in the UK and Ireland is taught at (Honours) degree level and three years of clinical experience are required, whereas in most other countries where nurse prescribing was or is being introduced, nurses are trained at Master degree level. The number of years of clinical experience required is also higher in some countries, for example in New Zealand and Australia, where the limit is set at four and five years respectively. As Abbott states, internal and external forces shape professional competition over jurisdiction. In the UK and Ireland the emphasis was on enhancing efficiency when introducing nurse prescribing, i.e. striving for quicker and more efficient patient access to medicines and better use of health professionals' skills and knowledge. In other countries, however, more urgent internal needs such as a shortage of physicians and unmet medication needs of patients in remote areas were the most important reasons for introducing nurse prescribing. Forces focussing on efficiency seem to lead to more extensive prescribing rights, at least for nurses in Ireland and the UK. This would appear to confirm Abbott's assumption that external and internal forces shape professional competition over jurisdiction. However, because of our focus on nurse prescribing, alternatives to prescribing, such as statutory exemptions and emergency provisions, were mainly left out of this review. Nevertheless, their possible presence across countries might have influenced the conditions under which nurse prescribing was realized as well, in addition to the influence of the internal and external forces we examined.

Perhaps the question as to whether or not national medical associations support the nurse prescribing initiative is also important when it comes to nurses' prescriptive authority. It is established that the British Medical Association in the UK has supported the nurse prescribing initiative from the outset [85] and this may have been beneficial to its extensive roll out. By contrast, in Australia, Spain and the USA, professional medical organizations have mainly opposed nurse prescribing [5,85,96], which may equally explain the relatively limited prescribing rights of US nurses, especially in view of the much longer period of familiarity with nurse prescribing in the USA compared to the UK.

However, on the basis of current data no definitive conclusions can be drawn about underlying mechanisms that operate between the forces that led to the introduction of nurse prescribing internationally and the scope of prescribing rights nurses enjoy. It would be interesting to further examine these mechanisms, preferably in a quantitative manner. Data on the percentage of total healthcare expenditure on medicines, number of physicians per capita and time of introduction of nurse prescribing could for example be used in an ecological analysis.

### Gaps in the literature

An interesting finding in this review is the near absence in the literature of reference to practice-related and organizational conditions under which nurses are allowed to prescribe medicines. This hinders a comparison and further theoretical interpretation of the organization of nurse prescribing internationally. For example, even though we found that most countries have mandatory registration systems in place for nurse prescribers, it remains unclear whether all nurses have individually registered provider numbers. However, where prescribing has been introduced to improve cost-effectiveness, individual provider numbers are needed to thoroughly monitor who prescribes which medicines how often and ascertain whether the implementation of nurse prescribing has had its intended effect.

When it comes to financial issues, likewise, many questions remain unanswered in the literature. What became clear however, is that reimbursement issues are not always properly catered for and this can, even with an otherwise good organisation, have far-reaching consequences for the success of nurse prescribing. For example, where medicines prescribed by nurses are not (fully) covered by insurance providers and/or national health programs, such as in some American states, this can generate an unfavourable reaction from the public towards nurse prescribing. Patients will prefer their physician to write their prescriptions, as reimbursement issues for this profession are well arranged. Consequently nurses might lose part of their workplace jurisdiction to the medical profession, who in their turn will claim more legal jurisdiction. Moreover, the fact that nurses' prescriptions are not always eligible for reimbursement underlines once more the full jurisdiction that medicine still has over prescribing, despite nurses' (limited) independent prescribing rights.

While we do not say that the organizational conditions have not been properly addressed across countries, they are largely missing from the literature. Both for interpreting the organization of nurse prescribing on a theoretical basis and for critically monitoring whether expected goals are being met, it is important that organizational conditions - as much as educational and legal conditions - are extensively discussed in the nurse prescribing literature.

### Limitations

It could be argued that this systematic review does not give a complete picture of the state of the art, as a number of policy documents and other relevant grey literature might potentially have been excluded from the review by our choice of search strategy. We choose this strategy, however, to safeguard the quality of sources. Even though the number of references to the organizational conditions under which nurses prescribe medicines as identified in this review proved somewhat disappointing, it is unlikely that this is due to our search strategy, as the educational and legal conditions under which nurses are allowed to prescribe medicines were sufficiently addressed in the identified literature.

Furthermore, as nurse prescribing is still in the process of development, there is a possibility that some of the included literature may be out of date in certain respects or doesn't contain the most recent developments in nurse prescribing. We tried to prevent this by including only publications from 2005 onwards that discussed the legal, educational and organizational conditions under which nurses are allowed to prescribe medicines. Nevertheless, it might prove beneficial to conduct a further survey among relevant stakeholders across all Western European and Anglo-Saxon countries that have realised or initiated nurse prescribing. This might also shed light on information that was largely missing from the scientific and professional literature, such as the organizational conditions under which nurse prescribing has been or will be realised internationally.

### Challenges for future research

Future research should provide more insight into the organizational and more especially the financial conditions under which nurses prescribe. These are not only important in everyday practice but are also indicators for the potential efficiency of nurse prescribing. There is also a need for more theory-based research on nurse prescribing. For example, we do not know how nurses' legal and workplace jurisdictions over prescribing relate to each other once legal prescriptive authority is obtained. There are indications that qualified nurse prescribers in the UK are not (fully) using their legal prescribing rights on the work floor, partly because of their own uncertainty about their educational preparation and partly resulting from organizational conditions such as a lack of system change within their work environment [36]. Future research should address this discrepancy between obtained legal authority and workplace jurisdiction. It is important to examine which mechanisms and forces influence this relationship.

## Conclusions

A diversity of external and internal forces has led to the introduction of nurse prescribing internationally. The precise nature of legal, educational and organizational conditions for nurse prescribing varies considerably, from situations where nurses prescribe independently to situations in which prescribing by nurses is only allowed under strict conditions and close supervision by physicians. As a result, a variety of jurisdictional settlements between the nursing and medical professions concerning the task of prescribing can be discerned. In some countries, nurses share (full) jurisdiction with the medical profession, whereas in others nurses prescribe in a subordinate position. However, in most countries the jurisdiction over prescribing remains predominantly with the medical profession. There seems to be an underlying mechanism linking the jurisdictional settlements between professions with the forces that led to the introduction of nurse prescribing. Forces focussing on efficiency appear to lead to more extensive prescribing rights.

## Competing interests

The authors declare that they have no competing interests.

## Authors' contributions

All authors made a substantive contribution to all parts of this study. MK developed and performed literature searches, was the primary responsible person for study inclusion, conducted the data-extraction and synthesis and compiled the first draft of this manuscript. AF and LvD participated in study inclusion and data-extraction and contributed substantively to drafting and revising the manuscript. PP participated in drafting and revising the manuscript. All authors read and approved the final manuscript.

## Pre-publication history

The pre-publication history for this paper can be accessed here:

http://www.biomedcentral.com/1472-6963/11/127/prepub

## Supplementary Material

Additional file 1 **Search strategies**. Click here for file

Additional file 2 **Characteristics of included publications**. Click here for file

Additional file 3 **Results verification search with relevant stakeholders in Western European and Anglo-Saxon countries**. Click here for file

Additional file 4 **Description of nurse prescribing in nine Western European and Anglo-Saxon countries according to core themes**. Click here for file

## References

[B1] Aarts J Koppel R Implementation of computerized physician order entry in seven countries Health Affairs 2009 28 404 414 10.1377/hlthaff.28.2.404 19275996

[B2] Drennan J Naughton C Allen D Hyde A Felle P O'Boyle K Treacy P Butler M National Independent Evaluation of the Nurse and Midwife Prescribing Initiative 2009 Dublin, University College Dublin

[B3] Jones A Nurse Prescribing in Mental Health 2009 Chichester: Wiley-Blackwell

[B4] van Ruth L Francke AL Mistiaen P Effects of nurse prescribing of medication: a systematic review Internet Journal of Healthcare Administration 2008 5 lit

[B5] Ball J Implementing nurse prescribing: an updated review of current practice internationally 2009 Geneve: International Council of Nurses

[B6] Buchan J Calman L Implementing nurse prescribing: an updated review of current practice internationally 2004 Geneve: International Council of Nurses

[B7] Abbott A The System of Professions: An Essay on the Division of Expert Labor 1988 Chicago: The University of Chicago Press

[B8] Buckley P Grime J Blenkinsopp A Inter- and intra- professional perspectives on non-medical prescribing in an NHS trust Pharmaceutical Journal 2006 277 394 398

[B9] Goundrey-Smith S Hannah KJ, Ball MJ Electronic Medicines Management and Non-Medical Prescribing Principles of Electronic Prescribin 2008 London: Springer-Verlag 119 136

[B10] Vrijhoef HJM Is It Justifiable to Treat Chronic Patients by Nurse Specialists? Evaluation of Effects on Quality of Care 2002 Maastricht: Universitaire Pers Maastricht

[B11] Sampson DA Sullivan-Marx EM, McGivern DO, Fairman JA, Greenberg SA The Idiosyncratic Politics of Prescriptive Authority: Comparing Two States' Legislative Negotiations Nurse Practitioners: The Evolution and Future of Advanced Practice 2010 New York: Springer Publishing Company 149 158

[B12] Department of Health Nurse prescribing FAQ 2010 http://webarchive.nationalarchives.gov.uk/+/www.dh.gov.uk/en/Healthcare/Medicinespharmacyandindustry/Prescriptions/TheNon-MedicalPrescribingProgramme/Nurseprescribing/DH_4123003

[B13] Watterson A Turner F Coull A Murray I An Evaluation of the Expansion of Nurse Prescribing in Scotland 2009 Edinburgh, Scottish Government Social Research

[B14] National Nursing and Nursing Education Taskforce National Nurse Prescribing Glossary 2006 Melbourne, National Nursing & Nursing Education Taskforce

[B15] Department of Health Supplementary prescribing FAQ 2010 http://webarchive.nationalarchives.gov.uk/+/www.dh.gov.uk/en/Healthcare/Medicinespharmacyandindustry/Prescriptions/TheNon-MedicalPrescribingProgramme/Supplementaryprescribing/DH_4123034

[B16] Hartley J Nurse Prescribing The Big Picture Nursing Times 2003 99 22 25 12718279

[B17] Department of Health Patient Group Directions 2010 http://www.dh.gov.uk/en/Publicationsandstatistics/Publications/PublicationsPolicyAndGuidance/Browsable/DH_4898318

[B18] Royal College of Nursing Patient Group Directions: Guidance and information for nurses 2004 London, Royal College of Nursing

[B19] Harris J Taylor J Mackie C Research literature review on prescribing 2004 Edinburgh, Scottish Executive Social Research

[B20] Ryan T Cash K Hannis D Nurse prescribing and in-patient alcohol detoxification Journal of Substance Use 1999 4 133 141

[B21] Allsop A Brooks L Bufton L Carr C Courtney Y Dale C Pittard S Thomas C. Supplementary prescribing in mental health and learning disabilities Nurs Stand 2005 19 54 58 15835438 10.7748/ns2005.04.19.30.54.c3836

[B22] Asher JB The case for nurse prescribing Nurs N Z 2005 11 17 16529279

[B23] Astles J Extended nurse prescribing: improving care for older people Br J Nurs 2006 15 150 151 16493322 10.12968/bjon.2006.15.3.20513

[B24] Baird A Independent and supplementary prescribing and PGDs Nursing Standard 2005 19 51 56 16161516 10.7748/ns2005.08.19.51.51.c3945

[B25] Barlow M Magorrian K Jones MA Edwards K Nurse prescribing in an Alzheimer's disease service: a reflective account Mental Health Practice 2008 11 32 35

[B26] Barrowman LM Review of the Implementation of the Nurse Prescribing Role 2007 Trust Nurses Association in Northern Ireland

[B27] Beckwith S Franklin P Oxford handbook of nurse prescribing 2007 Oxford: Oxford University Press

[B28] Berry PH Dahl JL Advanced practice nurse controlled substances prescriptive authority: a review of the regulations and implications for effective pain management at end-of-life Journal of Hospice & Palliative Nursing 2007 9 238 245 10.1097/01.NJH.0000289654.14752.1c21626670

[B29] Betts H Burgess J A preliminary evaluation of the first e-learning nurse prescribing course in England Stud Health Technol Inform 2006 122 153 157 17102238

[B30] Birkholz G Walker D Strategies for state statutory language changes granting fully independent nurse practitioner practice Nurse Pract 1994 19 54 58 8139802 10.1097/00006205-199401000-00004

[B31] Bowden L The impact of nurse prescribing on the role of the district nurse Nurse Prescribing 2005 3 79 86

[B32] Bradley E Nolan P Non-medical prescribing and mental health nursing: prominent issues Mental Health Practice 2005 8 16 19

[B33] Bradley E Campbell P Nolan P Nurse prescribers: who are they and how do they perceive their role? J Adv Nurs 2005 51 439 448 10.1111/j.1365-2648.2005.03527.x 16098160

[B34] Bradley E Blackshaw C Nolan P Nurse lecturers' observations on aspects of nurse prescribing training Nurse Educ Today 2006 26 538 544 10.1016/j.nedt.2006.01.008 16529849

[B35] Bradley E Hynam B Nolan P Nurse prescribing: reflections on safety in practice Soc Sci Med 2007 65 599 609 10.1016/j.socscimed.2007.03.051 17482332

[B36] Bradley E Wain P Nolan P Putting mental health nurse prescribing into practice Nurse Prescribing 2008 6 15 19

[B37] Bramley I Identifying future prescribers: taking an organizational approach Nurse Prescribing 2006 4 165 168

[B38] Bray K Dawson D Gibson V Howells H Cooper H McCormick J Plowright C. British Association of Critical Care Nurses position statement on prescribing in critical care Nursing in Critical Care 2009 14 224 234 10.1111/j.1478-5153.2009.00343.x 19706073

[B39] Brimblecombe N Parr AM Gray R Medication and mental health nurses: developing new ways of working Mental Health Practice 2005 8 12 14

[B40] Brookes D Smith A Non-Medical Prescribing in Healthcare Practice: A Toolkit for Students and Practitioners 2006 Basingstoke: Palgrave Macmillan

[B41] Bullough B Prescribing authority for nurses Nurs Econ 1983 1 122 125 6556443

[B42] Camp J Public policy implementation of nurse prescribing Nurse Prescribing 2008 6 252 257

[B43] Carey N Courtenay M Burke J Supplementary nurse prescribing for patients with skin conditions: a national questionnaire survey J Clin Nurs 2007 16 1230 1237 10.1111/j.1365-2702.2007.01641.x 17584340

[B44] Carey N Courtenay M An exploration of the continuing professional development needs of nurse independent prescribers and nurse supplementary prescribers who prescribe medicines for patients with diabetes Journal of Clinical Nursing 2010 19 208 216 10.1111/j.1365-2702.2009.02943.x 20500258

[B45] Carey N Stenner K Courtenay M Adopting the prescribing role in practice: exploring nurses' views in a specialist children's hospital Paediatric Nursing 2009 21 25 29 19947105 10.7748/paed2009.11.21.9.25.c7357

[B46] Cooper R Guillaume L Avery T Anderson C Bissell P Hutchinson A Lymn J Murphy E Ward P Ratcliffe J. Nonmedical prescribing in the United kingdom: developments and stakeholder interests J Ambul Care Manage 2008 31 244 252 18574383 10.1097/01.JAC.0000324670.91153.b4

[B47] Cooper R Anderson C Avery T Bissell P Guillaume L Hutchinson A Lymn J Murphy E Ratcliffe J Ward P. Stakeholders' views of UK nurse and pharmacist supplementary prescribing J Health Serv Res Policy 2008 13 215 221 10.1258/jhsrp.2008.008004 18806179

[B48] Courtenay M Carey N Extended prescribing power in diabetes: a landmark for nurses Diabetes & Primary Care 2006 8 97 10021626717

[B49] Courtenay M Prescribing 2007 Journal of Community Nursing 2007 21 11 13

[B50] Courtenay M Nurse prescribing, policy, practice and evidence base Br J Community Nurs 2008 13 563 566 19060833 10.12968/bjcn.2008.13.12.31830

[B51] Courtenay M Carey N Nurse prescribing by children's nurses: views of doctors and clinical leads in one specialist children's hospital J Clin Nurs 2009 18 2668 2675 10.1111/j.1365-2702.2009.02799.x 19619207

[B52] Courtenay M Nurse prescribing in primary care: where are we now? Nursing in Practice: The Journal for Today's Primary Care Nurse 2009

[B53] Courtenay M Stenner K Carey N Nurses' and doctors' views about the prescribing programme Nurse Prescribing 2009 7 412 417

[B54] Craig EJ A review of prescriptive authority for nurse practitioners J Perinat Neonatal Nurs 1996 10 29 35 8717769 10.1097/00005237-199606000-00005

[B55] Creedon R O'Connell E Introducing nurse prescribing: an Irish perspective Nurse Prescribing 2009 7 507 511

[B56] Culley F Understanding developments in non-medical prescribing Nurs Times 2005 101 30 33 16149702

[B57] Daly G Non-medical prescribing: A discussion on practice implications Work Based Learn Prim Care 2006 4 236 242

[B58] Donato AS Nurse practitioners in Holland: Definition, preparation, and prescriptive authority J Am Acad Nurse Pract 2009 21 585 587 10.1111/j.1745-7599.2009.00452.x 19900219

[B59] Faucher MA Prescriptive authority for advanced nurse practitioners: a blue-print for action J Pediatr Health Care 1992 6 25 31 10.1016/0891-5245(92)90062-9 1545328

[B60] Fisher R Relationships in nurse prescribing in district nursing practice in England: a preliminary investigation Int J Nurs Pract 2005 11 102 107 10.1111/j.1440-172X.2005.00513.x 15853788

[B61] Forchuk C Kohr R Prescriptive authority for nurses: the Canadian perspective Perspect Psychiatr Care 2009 45 3 8 10.1111/j.1744-6163.2009.00194.x 19154247

[B62] Ford K Otway C Health visitor prescribing: the need for CPD Nurse Prescribing 2008 6 387 403

[B63] Gallagher J O'Gara C Sessay M Luty J Nurse prescribing in addiction services: client benefits Nurs Stand 2006 20 42 44 16922291 10.7748/ns2006.08.20.48.42.c4480

[B64] Gilmour CE Bickford J Grundy-Bowers M, Davies J Patient Group Directions and Nurse Prescribing Advanced Clinical Skills for GU Nurses 2007 Chichester: John Wiley & Sons

[B65] Goswell N Siefers R Experiences of ward-based nurse prescribers in an acute ward setting Br J Nurs 2009 18 34 37 19127229 10.12968/bjon.2009.18.1.32087

[B66] Grassby P Prescribing controlled drugs Practice Nurse 2005 30 24 27

[B67] Gray R Parr AM Brimblecombe N Mental health nurse supplementary prescribing: Mapping progress 1 year after implementation Psychiatr Bull 2005 29 295 297 10.1192/pb.29.8.295

[B68] Green A Westwood O Smith P Peniston-Bird F Holloway D Provision of continued professional development for non-medical prescribers within a South of England Strategic Health Authority: a report on a training needs analysis J Nurs Manag 2009 17 603 614 10.1111/j.1365-2834.2008.00892.x 19575719

[B69] Greveson K An audit of independent non-medical prescribing in inflammatory bowel disease Gastrointestinal Nursing 2009 7 23 27

[B70] Griffith R The role of nurse prescribers in the management of controlled drugs Nurse Prescribing 2006 4 155 160

[B71] Griffith R Legal requirements for the prescribing and administration of medicines Br J Community Nurs 2007 12 477 481 18073650 10.12968/bjcn.2007.12.10.27288

[B72] Hall J Supplementary prescribing for nurses Br J Nurs 2005 14 968 70, 72 16237351 10.12968/bjon.2005.14.18.19883

[B73] Hall J Cantrill J Noyce P Why don't trained community nurse prescribers prescribe? J Clin Nurs 2006 15 403 412 10.1111/j.1365-2702.2006.01227.x 16553753

[B74] Hansen-Turton T Ritter A Valdez B Developing alliances: how advanced practice nurses became part of the prescription for Pennsylvania Policy Polit Nurs Pract 2009 10 7 15 19383617 10.1177/1527154408330206

[B75] Harkless GE Prescriptive authority: debunking common assumptions Nurse Pract 1989 14 57 1 2671826

[B76] Hemingway S Ely V Prescribing by mental health nurses: the UK perspective Perspect Psychiatr Care 2009 45 24 35 10.1111/j.1744-6163.2009.00197.x 19154250

[B77] Hinchliffe AL Supplementary Prescribing in Wales Nurse Prescriber 2006 1

[B78] Hobden A An outline of the current options for the supply and administration of medicines British Journal of Neuroscience Nursing 2007 3 313 317

[B79] Hughes F Lockyer H Evidence and engagement in the introduction of nurse prescribing in New Zealand Nurse Prescribing 2004 2 131 136

[B80] Jacobs SH Boddy JM The genesis of advanced nursing practice in New Zealand: policy, politics and education Nurs Prax N Z 2008 24 11 22 18557367

[B81] Jones A Supplementary prescribing: potential ways to reform hospital psychiatric care J Psychiatr Ment Health Nurs 2006 13 132 138 10.1111/j.1365-2850.2006.00898.x 16608467

[B82] Jones A Supplementary prescribing: relationships between nurses and psychiatrists on hospital psychiatric wards J Psychiatr Ment Health Nurs 2006 13 3 11 10.1111/j.1365-2850.2006.00888.x 16441387

[B83] Jones A Exploring independent nurse prescribing for mental health settings J Psychiatr Ment Health Nurs 2008 15 109 117 10.1111/j.1365-2850.2007.01216.x 18211558

[B84] Jones K Developing a prescribing role for acute care nurses Nurs Manag 2009 16 24 28 10.7748/nm2009.11.16.7.24.c7351 19943411

[B85] Jones M Nurse prescribing: politics to practice 1999 1 Edinburgh: Bailliere Tindall & Royal College of Nursing

[B86] Koch LW Pazaki SH Campbell JD The first 20 years of nurse practitioner literature: an evolution of joint practice issues Nurse Pract 1992 17 62 6 68,71 10.1097/00006205-199208000-00017 1542464

[B87] Latter S Safety and quality in independent prescribing: an evidence review Nurse Prescribing 2008 6 59 66

[B88] Lee GA Fitzgerald L A clinical internship model for the nurse practitioner programme Nurse Educ Pract 2008 8 397 404 10.1016/j.nepr.2008.03.002 18472301

[B89] Lilley M Marshall J McIntosh N Bisland K McNeill E Morton E Independent nurse prescribing in an acute hospital setting Paediatr Nurs 2005 17 14 18 15906893 10.7748/paed2005.05.17.4.14.c985

[B90] Manchester A Nurses gain right to prescribe Nurs N Z 1998 4 12 10586752

[B91] Meadows A Sheehan NJ Prescribing and injecting: the expanding role of the rheumatology nurse Musculoskeletal Care 2005 3 176 178 10.1002/msc.7 17042006

[B92] O'Hare G The development of Nurse Prescribing within the Cancer Nursing Team of a district general hospital Scottish Nurse 2007 10 24 25

[B93] Padmore E Nurse prescribing in diabetes care Diabet Med 2005 22 Suppl 1 18 20 15610152 10.1111/j.1464-5491.2005.1531h.x

[B94] Patel MX Robson D Rance J Ramirez NM Memon TC Bressington D Gray R. Attitudes regarding mental health nurse prescribing among psychiatrists and nurses: a cross-sectional questionnaire study Int J Nurs Stud 2009 46 1467 1474 10.1016/j.ijnurstu.2009.04.010 19482282

[B95] Peniston-Bird F Key Questions - Non-medical Prescribing Nursingtimes.net 2007

[B96] Plonczynski D Oldenburg N Buck M The past, present and future of nurse prescribing in the United States Nurse Prescribing 2003 1 170

[B97] Pollock L Dudgeon N The Scottish Nurse Prescribing Audit 2006 2006

[B98] Pontin D Jones S Children's nurses and nurse prescribing: a case study identifying issues for developing training programmes in the UK J Clin Nurs 2007 16 540 548 10.1111/j.1365-2702.2006.01585.x 17335530

[B99] Ring M Implementing nurse prescribing - the challenges Nurse Prescriber 2006 1

[B100] Ross J Researching the barriers to mental health nurse prescribing Nurse Prescribing 2009 7 249 253

[B101] Ryan-Woolley B McHugh G Luker K Exploring the views of nurse prescribing among Macmillan nurses Br J Community Nurs 2008 13 171 177 18595310 10.12968/bjcn.2008.13.4.29026

[B102] Ryan N Nurse prescribing in child and adolescent mental health services Mental Health Practice 2007 10 35 37

[B103] Shuttleworth A Prescriber. Are nurses ready to take on the BNF? Nursing Times 2005 101 56 57 16350523

[B104] Skingsley D Bradley EJ Nolan P Neuropharmacology and mental health nurse prescribers J Clin Nurs 2006 15 989 997 10.1111/j.1365-2702.2006.01378.x 16879543

[B105] Snowden AW Nurse prescribing in mental health Nurs Stand 2006 20 41 46 16605231 10.7748/ns2006.03.20.29.41.c4106

[B106] Snowden AWA Nurse prescribing. Exploring the impact of mental health nurse prescribing British Journal of Nursing (BJN) 2006 15 1114 1118 10.12968/bjon.2006.15.20.22296 17170660

[B107] Spence D Anderson M Implementing a prescribing practicum within a Master's degree in advanced nursing practice Nurs Prax N Z 2007 23 27 42 18293655

[B108] Stenner K Courtenay M The role of inter-professional relationships and support for nurse prescribing in acute and chronic pain J Adv Nurs 2008 63 276 283 10.1111/j.1365-2648.2008.04707.x 18702774

[B109] Stenner K Carey N Courtenay M Nurse prescribing in dermatology: doctors' and non-prescribing nurses' views J Adv Nurs 2009 65 851 859 10.1111/j.1365-2648.2008.04944.x 19243463

[B110] Strickland-Hodge B Nurse prescribing: the elephant in the room? Qual Prim Care 2008 16 103 107 18700087

[B111] Tarmina MS How Utah NPs obtained prescriptive privileges Nurse Pract 1982 7 9 12 7121903

[B112] Ulfvarson J Mejyr S Bergman U Nurses are increasingly involved in pharmacovigilance in Sweden Pharmacoepidemiol Drug Saf 2007 16 532 537 10.1002/pds.1336 17072915

[B113] Warburton P Kahn P Improving the numeracy skills of nurse prescribers Nurs Stand 2007 21 40 43 17436893 10.7748/ns2007.03.21.28.40.c4534

[B114] Warner D Theory of nurse prescribing Journal of Community Nursing 2005 19 12

[B115] Wells J Bergin M Gooney M Jones A Views on nurse prescribing: a survey of community mental health nurses in the Republic of Ireland J Psychiatr Ment Health Nurs 2009 16 10 17 10.1111/j.1365-2850.2008.01322.x 19192081

[B116] Wilkinson K Supplementary prescribing for overactive bladder Nurs Stand 2005 19 38 42 15700865 10.7748/ns2005.01.19.19.38.c3788

[B117] Peet Rvd De zelfstandige bevoegdheid van de verpleegkundig specialist [The independent authority of the Nurse Specialist] Tijdschrift voor Verpleegkundigen 2010 120 45 49

[B118] Peet Rvd De voorschrijfbevoegdheid van verpleegkundigen [The prescriptive authority of nurses] Tijdschrift voor Verpleegkundigen 2010 120 50 53

[B119] Houweling ST Kleefstra N Hateren KJJv Kooy A Groenier KH Ten Vergert E Meyboom-de Jong B Bilo HJG. Diabetes specialist nurse as main care provider for patients with type 2 diabetes The Netherlands Journal of Medicine 2010 67 279 284 19687522

[B120] Beekman E Patterson L Nurse prescribing in New Zealand: professional gain or political loss? Nursing Praxis in New Zealand 2003 19 15 22

[B121] Berry D Courtenay M Bersellini E Attitudes towards, and information needs in relation to, supplementary nurse prescribing in the UK: an empirical study J Clin Nurs 2006 15 22 28 10.1111/j.1365-2702.2005.01258.x 16390520

[B122] Chaston D Seccombe J Mental health nurse prescribing in New Zealand and the United kingdom: comparing the pathways Perspect Psychiatr Care 2009 45 17 23 10.1111/j.1744-6163.2009.00196.x 19154249

[B123] Cooper RJ Anderson C Avery T Bissell P Guillaume L Hutchinson A James V Lymn J McIntosh A Murphy E Ratcliffe J Read S Ward P. Nurse and pharmacist supplementary prescribing in the UK--a thematic review of the literature Health Policy 2008 85 277 292 10.1016/j.healthpol.2007.07.016 17900744

[B124] Courtenay M Carey N Burke J Independent extended supplementary nurse prescribers, their prescribing practice and confidence to educate and assess prescribing students Nurse Educ Today 2007 27 739 747 10.1016/j.nedt.2006.10.007 17137684

[B125] Courtenay M Carey N Preparing nurses to prescribe medicines for patients with diabetes: a national questionnaire survey J Adv Nurs 2008 61 403 412 10.1111/j.1365-2648.2007.04534.x 18234038

[B126] Courtenay M Carey N The prescribing practices of nurse independent prescribers caring for patients with diabetes: Findings from a national questionnaire survey Pract Diabetes Int 2008 25 152 157 10.1002/pdi.1235

[B127] David A Brown E How Swedish nurses are tackling nurse prescribing Nurs Times 1995 91 23 24 8559677

[B128] Davis K Drennan V Evaluating nurse prescribing behaviour using constipation as a case study Int J Nurs Pract 2007 13 243 253 10.1111/j.1440-172X.2007.00634.x 17640246

[B129] Dragon N A new prescription needed for nurse practitioners. (Cover story) Australian Nursing Journal 2008 16 20 23 18841716

[B130] Durand T The prescriptive emergency nurse practitioner: an analysis to substantiate ENPs' inclusion within current legislative proposals Accid Emerg Nurs 1998 6 110 114 10.1016/S0965-2302(98)90010-8 9677881

[B131] Elsom S Happell B Manias E Nurse practitioners and medical practice: opposing forces or complementary contributions? Perspect Psychiatr Care 2009 45 9 16 10.1111/j.1744-6163.2009.00195.x 19154248

[B132] Evans D Mental Health Nurse Prescribing: Challenges in Theory and Practice Mental Health and Learning Disabilities Research and Practice 2009 6 97 106

[B133] Haidar E Role of the advanced nurse practitioner in prescribing and general practice Nurse Prescribing 2007 5 74 78

[B134] Hemingway S McAllister M Bailey K Coates K Mitchell S Fenwick M Focus On...Nurse Prescribing in Mental Health Care: What can the USA teach us? Nurse Prescriber 2006 1

[B135] Jones A Jones M Mental health nurse prescribing: issues for the UK J Psychiatr Ment Health Nurs 2005 12 527 535 10.1111/j.1365-2850.2005.00857.x 16164502

[B136] Latter S Maben J Myall M Young A Evaluating nurse prescribers' education and continuing professional development for independent prescribing practice: findings from a national survey in England Nurse Educ Today 2007 27 685 696 10.1016/j.nedt.2006.10.002 17123668

[B137] Latter S Maben J Myall M Young A Baileff A Evaluating prescribing competencies and standards used in nurse independent prescribers' prescribing consultations Journal of Research in Nursing 2007 12 7 26 10.1177/1744987106073949

[B138] Lim AG Honey M Kilpatrick J Framework for teaching pharmacology to prepare graduate nurse for prescribing in New Zealand Nurse Educ Pract 2007 7 348 353 10.1016/j.nepr.2006.11.006 17689462

[B139] Lockwood EB Fealy GM Nurse prescribing as an aspect of future role expansion: the views of Irish clinical nurse specialists J Nurs Manag 2008 16 813 820 10.1111/j.1365-2934.2008.00853.x 19017243

[B140] Lymn J Bath-Hextall F Wharrad H Pharmacology education for nurse prescribing students - a lesson in reusable learning objects BMC Nurs 2008 7 10.1186/1472-6955-7-2 PMC2263034 18215261

[B141] Sheer B Wong FK The development of advanced nursing practice globally J Nurs Scholarsh 2008 40 204 211 10.1111/j.1547-5069.2008.00242.x 18840202

[B142] Snowden A The history of prescribing Nurse Prescribing 2008 6 530 537

