# Supplementary material for: Nurse prescribing of medicines in Western European and Anglo-Saxon countries: a systematic review of the literature
Source: BMC Health Serv Res. 2011 May 27;11:127. doi: 10.1186/1472-6963-11-127 (PMC3141384; doi:10.1186/1472-6963-11-127)
Supplement: Additional file 1 — Search strategies. [file 1472-6963-11-127-S1.DOC]

**Additional file 1 – Search strategies**

***Databases***

**Search strategy Academic Search Elite dd. 06-01-2010**

| **Search ID#** | **Search Terms** | **Search Options** | **Results** |
| --- | --- | --- | --- |
| S21 | TI nurs* and AB nurs* | Boolean/Phrase | 31788 |
| S22 | TI prescri* and AB prescri* | Boolean/Phrase | 4370 |
| S23 | DE "NURSES" (exploded) | Boolean/Phrase | 32498 |
| S24 | TI formulary and AB formulary | Boolean/Phrase | 123 |
| S25 | DE "DRUGS -- Prescribing" (exploded) | Boolean/Phrase | 3732 |
| S26 | (S23 and S25) | Boolean/Phrase | 222 |
| S27 | "nurse prescribing" | Boolean/Phrase | 311 |
| S28 | (S21 and S22) | Boolean/Phrase | 277 |
| S29 | (S23 and S24) | Boolean/Phrase | 3 |
| S30 | (S26 or S27 or S28 or S29) | Boolean/Phrase | 542 |

542 references were included in the original literature list.

**Search strategy CINAHL dd. 06-01-2010**

| **Search ID#** | **Search Terms** | **Search Options** | **Results** |
| --- | --- | --- | --- |
| S1 | TI nurs* and AB nurs* | Boolean/Phrase | 61367 |
| S2 | TI prescri* and AB prescri* | Boolean/Phrase | 2557 |
| S3 | (MH "Nurses+") | Boolean/Phrase | 110856 |
| S4 | TI formulary and AB formulary | Boolean/Phrase | 71 |
| S5 | (MH "Prescriptions, Drug") | Boolean/Phrase | 2610 |
| S6 | (MH "Prescriptions, Drug") and (MH "Nurses+") | Boolean/Phrase | 141 |
| S7 | "nurse prescribing" | Boolean/Phrase | 727 |
| S8 | (S1 and S2) | Boolean/Phrase | 509 |
| S9 | (S3 and S4) | Boolean/Phrase | 1 |
| S10 | (S6 or S7 or S8 or S9) | Boolean/Phrase | 1067 |

1067 references were included in the original literature list.

**Search strategy EMBASE dd. 22-12-2009**

| **No.** | **Search query** | **Results** |
| --- | --- | --- |
| 1 | nurs*:ab,ti AND [embase]/lim | 77087 |
| 2 | prescri*:ab,ti AND [embase]/lim | 85396 |
| 3 | 'nurse'/exp AND [embase]/lim | 20665 |
| 4 | formulary:ab,ti AND [embase]/lim | 2263 |
| 5 | 'nurse'/exp AND 'prescription'/exp AND [embase]/lim | 826 |
| 6 | 'nurse prescribing' AND [embase]/lim | 72 |
| 7 | #1 AND #2 AND [embase]/lim | 2777 |
| 8 | #3 AND #4 AND [embase]/lim | 31 |
| 9 | #5 OR #6 OR #7 OR #8 AND [embase]/lim | 3207 |

3207 references were included in the original literature list.

**Search strategy NIVEL Catalogue dd. 04-01-2010**

| **No.** | **Search query** | **Results** |
| --- | --- | --- |
| 1 | nurs* AND prescri* | 43 |
| 2 | nurs* AND formulary | 1 |
| 3 | nurs* AND drug prescription | 2 |
| 4 | nurse prescribing | 29 |

After removing duplicates 42 references were included in the original literature list**.**

**Search strategy PubM**ed dd. 22-12-2009

| **Search** | **Most Recent Queries** | **Results** |
| --- | --- | --- |
| #6 | Search **nurs* [tiab]** | 275043 |
| #7 | Search **prescri* [tiab]** | 90279 |
| #8 | Search **"Nurses"[Mesh]** | 59805 |
| #9 | Search **formulary [tiab]** | 2213 |
| #10 | Search **"Nurses"[Mesh] AND "Drug Prescriptions"[Mesh]** | 657 |
| #11 | Search **"nurse prescribing"** | 307 |
| #12 | Search **#6 AND #7** | 4306 |
| #13 | Search **#8 AND #9** | 31 |
| #14 | Search **#10 OR #11 OR #12 OR #13** | 4664 |

4664 references were included in the original literature list.

**Search strategy Web of Science dd. 24-12-2009**

| **No.** | **Search query** | **Results** |
| --- | --- | --- |
| 1 | TI=nurs* | 75785 |
| 2 | TI=prescri* | 24110 |
| 3 | TI=formulary | 967 |
| 4 | TS=(nurs* AND drug prescription) | 372 |
| 5 | TS="nurse prescribing" | 132 |
| 6 | #1 AND #2 | 349 |
| 7 | #1 AND #3 | 7 |
| 8 | #4 OR #5 OR #6 OR #7 | 721 |

721 references were included in the original literature list.

**Total result of literature searches in databases: 10243 references.**

***Websites***

| **Name, link and date searched** | **Search strategy** | **Number of references found** |
| --- | --- | --- |
| Escriber.com  <http://www.escriber.com/view/0/index.html>  04-01-2010 | Website was searched using basic search facilities. Search terms:  1. “nurse prescribing”  2. “independent (nurse) prescribing”  3. “autonomous prescribing”  4. “supplementary (nurse) prescribing”  5. “dependent (nurse) prescribing”  6. “collaborative prescribing”  7. “group protocols”  8. “patient group directions”  9. “nurse formulary” | N= 5  N= 3  –  N= 4  –  –  –  N= 2  –  After removing duplicates 12 references were included in the original literature list**.** |
| Google Scholar  <http://scholar.google.nl/>  05-01-2010 | Website searched using basic search facilities. Search term: “nurse prescribing”. | After removing duplicates 1000 references were included in the original literature list**.** |
| Internurse.com  <http://www.internurse.com/>  04-01-2010 | Website was searched using advanced search facilities. Search terms:  1. “nurse prescribing”  2. “independent (nurse) prescribing”  3. “autonomous prescribing”  4. “supplementary (nurse) prescribing”  5. “dependent (nurse) prescribing”  6. “collaborative prescribing”  7. “group protocols”  8. “patient group directions”  9. “nurse formulary” | N >500  N= 382  N= 1  N= 339  N= 30  N= 1  N= 56  N= 183  N= 31  After removing duplicates 690 references were included in the original literature list**.** |
| Nurse Prescriber  <http://www.nurse-prescriber.co.uk/>  04-01-2010 | Website was manually searched. | After removing duplicates 78 references were included in the original literature list**.** |
| Nursingtimes.net  <http://www.nursingtimes.net/>  04-01-2010 | Website was searched using basic search facilities. Search terms:  1. “nurse prescribing”  2. “independent (nurse) prescribing”  3. “autonomous prescribing”  4. “supplementary (nurse) prescribing”  5. “dependent (nurse) prescribing”  6. “collaborative prescribing”  7. “group protocols”  8. “patient group directions”  9. “nurse formulary” | N= 118  N= 40  –  N= 40  N= 2  –  N= 10  N= 44  N= 2  After removing duplicates 162 references were included in the original literature list. |
| Virginia Henderson International Nursing Library  <http://www.nursinglibrary.org/portal/main.aspx>  24-12-2009 | Website was searched using basic search facilities. Search terms:  1. “nurse prescribing”  2. “independent (nurse) prescribing”  3. “autonomous prescribing”  4. “supplementary (nurse) prescribing”  5. “dependent (nurse) prescribing”  6. “collaborative prescribing”  7. “group protocols”  8. “patient group directions”  9. “nurse formulary” | N= 52  N= 7  –  –  N= 9  N= 5  N= 129  N= 16  N= 7  After removing duplicates 185 references were included in the original literature list**.** |
| World Health Organisation  <http://www.who.int/en/>  18-01-2010 | Website was searched using advanced search facilities. Search terms:  1. “nurse prescribing”  2. “independent (nurse) prescribing”  3. “autonomous prescribing”  4. “supplementary (nurse) prescribing”  5. “dependent (nurse) prescribing”  6. “collaborative prescribing”  7. “group protocols”  8. “patient group directions”  9. “nurse formulary” | N= 38  N= 12  –  N= 4  –  N= 1  N= 2  N= 1  –  After removing duplicates 32 references were included in the original literature list**.** |

**Total result of literature searches in websites: 2159 references.**

**Total result literature searches**

Literature searches in databases: N= 10243 references

Literature searches in websites: N= 2159 references

Expert advice: N= 3 references

**Total result literature searches: N = 12405 references.**
