# Supplementary material for: Nurse prescribing of medicines in Western European and Anglo-Saxon countries: a systematic review of the literature
Source: BMC Health Serv Res. 2011 May 27;11:127. doi: 10.1186/1472-6963-11-127 (PMC3141384; doi:10.1186/1472-6963-11-127)
Supplement: Additional file 2 — Characteristics of included publications. [file 1472-6963-11-127-S2.DOC]

**Additional file 2 – Characteristics of included publications**

| **Author(s)** | **Year** | **Country/setting** | **Focus of publication** | **Model(s) of NP discussed** | **NP dimension(s) discussed[[1]](#endnote-2)** |
| --- | --- | --- | --- | --- | --- |
| Allsop [21] | 2005 | The United Kingdom | Supplementary prescribing in mental health and learning disabilities | Independent and supplementary nurse prescribing | FORC, LEGL, EDUC |
| Asher [22] | 2005 | New Zealand | The initiative to extend prescribing rights | Independent nurse prescribing | FORC, EDUC, PROR |
| Astles [23] | 2006 | The United Kingdom | The benefits to older people in a hospital setting through extended nurse prescribing. | Independent nurse prescribing | FORC, LEGL, PROR |
| Baird [24] | 2005 | The United Kingdom | The forms of prescribing and administration of medication for non-medical staff. | Independent and supplementary nurse prescribing, and patient group directions | FORC, LEGL, EDUC |
| Ball [5] | 2009 | Australia, Canada, Ireland, Netherlands, New Zealand, Spain, Sweden, the UK, the USA | The worldwide developments in nurse prescribing policy and practice | Independent and supplementary nurse prescribing, and patient group directions | FORC, LEGL, EDUC, PROR |
| Barlow, Magorrian, Jones et al. [25] | 2008 | The United Kingdom | Experiences of implementing nurse prescribing in a specialist dementia service. | Supplementary nurse prescribing | FORC, LEGL, EDUC |
| Barrowman [26] | 2007 | The United Kingdom (Northern Ireland) | The implementation of nurse prescribing in Northern Ireland. | Independent and supplementary nurse prescribing | FORC, LEGL, EDUC |
| Beckwith, Franklin [27] | 2007 | The United Kingdom | Practical guidance on all aspects of prescribing | Independent and supplementary nurse prescribing, and patient group directions | FORC, LEGL, EDUC, PROR |
| Beekman, Patterson [120] | 2003 | New Zealand, Australia | The drivers behind changes in nursing work and in particular nurse prescribing | Independent nurse prescribing | FORC, LEGL, EDUC |
| Berry, Courtenay, Bersellini [121] | 2006 | The United Kingdom, Sweden | Attitudes towards and information needs in relation to supplementary prescribing | Independent and supplementary nurse prescribing | FORC, LEGL |
| Berry, Dahl [28] | 2007 | The USA | Prescriptive authority of APNs in the USA | Independent nurse prescribing | FORC, LEGL, EDUC |
| Betts, Burgess [29] | 2006 | The United Kingdom | The evaluation of the first e-learning nurse prescribing course in England. | Independent and supplementary nurse prescribing | FORC, LEGL, EDUC, PROR |
| Birkholz, Walker [30] | 1994 | The USA (Alaska, New Mexico) | The scope of practice and prescriptive privileges of Nurse Practitioners | Independent nurse prescribing | FORC |
| Bowden [31] | 2005 | The United Kingdom | The experiences of nurse prescribing by a group of district nurses working within one of the UK pilot sites. | Independent and supplementary nurse prescribing | FORC, LEGL |
| Bradley, Nolan [32] | 2005 | The United Kingdom | The issues around non-medical prescribing that are currently prominent within mental health teams. | Independent and supplementary nurse prescribing | FORC, LEGL, EDUC |
| Bradley, Campbell, Nolan [33] | 2005 | The United Kingdom | The professional background and reasons for choosing to become nurse prescribers of recently qualified nurse prescribers. | Independent and supplementary nurse prescribing | FORC, LEGL, EDUC |
| Bradley, Blackshaw, Nolan [34] | 2006 | The United Kingdom | Nurse lecturers’ experiences of delivering nurse prescribing courses. | Independent and supplementary nurse prescribing | FORC, LEGL, EDUC |
| Bradley, Hynam, Nolan [35] | 2007 | The United Kingdom | The description, rating and safety of prescribing by recently qualified nurse prescribers. | Independent and supplementary nurse prescribing | FORC, LEGL |
| Bradley, Wain, Nolan [36] | 2008 | The United Kingdom | Why some nurses put their prescribing role in practice and others do not. | Independent and supplementary nurse prescribing | FORC |
| Bramley [37] | 2006 | The United Kingdom | The strategic approach to be taken by managers when identifying future nurse prescribers. | Independent and supplementary nurse prescribing | FORC, LEGL, EDUC |
| Bray, Dawson, Gibson et al. [38] | 2009 | The United Kingdom | The support and informing of nurses in critical care currently undertaking prescribing and those who are intending to prescribe. | Independent and supplementary nurse prescribing, and patient group directions | FORC, LEGL, EDUC |
| Brimblecombe, Parr, Gray [39] | 2005 | The United Kingdom | Development of new ways of working for mental health nurses | Supplementary nurse prescribing and patient group directions | FORC, LEGL, EDUC |
| Brookes, Smith [40] | 2006 | The United Kingdom | Non-medical prescribing in health care practice | Independent and supplementary nurse prescribing, and patient group directions | FORC, LEGL, EDUC, PROR |
| Buchan, Calman [6] | 2004 | Australia, Canada, New Zealand, Sweden, the UK, the USA | A review of current nurse prescribing practices | Independent and supplementary nurse prescribing, and patient group directions | FORC, LEGL, EDUC, PROR |
| Bullough [41] | 1983 | The USA | The prescribing authority for nurses in American states | Independent nurse prescribing | FORC |
| Camp [42] | 2008 | The United Kingdom (Scotland) | The implementation process of the public policy of nurse prescribing from a Scottish perspective. | Independent and supplementary nurse prescribing | FORC, LEGL, EDUC, PROR |
| Carey, Courtenay, Burke [43] | 2007 | The United Kingdom | The prescribing practices of nurses who prescribe for patients with skin conditions and related facilitating and inhibiting factors. | Independent and supplementary nurse prescribing | FORC, LEGL |
| Carey, Stenner, Courtenay [45] | 2009 | The United Kingdom | The views of children’s nurses on the adoption of the prescribing role in their practice. | Independent and supplementary nurse prescribing | FORC, LEGL |
| Carey, Courtenay [44] | 2010 | The United Kingdom | The pharmaceutical knowledge and provision of CPD to nurses who prescribe for patients with diabetes. | Independent and supplementary nurse prescribing | FORC, LEGL, EDUC |
| Chaston, Seccombe [122] | 2009 | The UK, New Zealand | The difference in educational preparation and context with regards to nurse prescribing between New Zealand and the UK. | Independent and supplementary nurse prescribing | FORC, EDUC |
| Cooper, Anderson, Avery et al. [123] | 2008 | The UK, the USA | Reviewing the literature on nurse and pharmacist SP to inform further research, policy and education. | Supplementary nurse prescribing | FORC, LEGL, EDUC |
| Cooper, Guillaume, Avery et al. [46] | 2008 | The United Kingdom | The developments and stakeholder interests of non medical prescribing. | Independent and supplementary nurse prescribing | FORC, LEGL, EDUC, PROR |
| Cooper, Anderson, Avery et al. [47] | 2008 | The United Kingdom | The views of stakeholders involved in SP on benefits, facilitators, challenges, and safety and costs. | Independent and supplementary nurse prescribing and patient group directions | FORC, LEGL, EDUC, PROR |
| Courtenay, Carey [48] | 2006 | The United Kingdom | The advancement of nurse independent- and supplementary prescribing | Independent and supplementary nurse prescribing | LEGL, EDUC |
| Courtenay, Carey, Burke [124] | 2006 | The UK, Sweden, Canada, Australia, the USA | The prescribing practice and confidence to educate and access prescribing students of nurse prescribers. | Independent and supplementary nurse prescribing | FORC, LEGL, EDUC |
| Courtenay [49] | 2007 | The United Kingdom | The progression of nurse prescribing in the UK | Independent and supplementary nurse prescribing | LEGL, EDUC |
| Courtenay, Carey [125] | 2007 | The UK, Sweden, Australia, New Zealand, the USA | The preparedness of nurses to prescribe medicines for patients with diabetes. | Independent and supplementary nurse prescribing | FORC, LEGL, EDUC, PROR |
| Courtenay, Carey [126] | 2008 | The UK, Sweden, Australia, Canada, the USA | The prescribing practices of nurse independent prescribers caring for patients with diabetes | Independent and supplementary nurse prescribing | LEGL, EDUC |
| Courtenay [50] | 2008 | The United Kingdom | Recent policy changes surrounding nurse prescribing and specifically changes pertinent to community nurses | Independent and supplementary nurse prescribing | FORC, LEGL, EDUC, PROR |
| Courtenay [52] | 2009 | The United Kingdom | Overview of nurse prescribing and the required education and training | Independent nurse prescribing | FORC, LEGL, EDUC |
| Courtenay, Carey [51] | 2009 | The United Kingdom | Views of doctors and clinical leads on nurse prescribing by children’s nurses | Independent and supplementary nurse prescribing | FORC, LEGL, EDUC |
| Courtenay, Stenner, Carey [53] | 2009 | The United Kingdom | The views of doctors and nurses who care for people with diabetes about the prescribing programme. | Independent and supplementary nurse prescribing | LEGL, EDUC |
| Craig [54] | 1996 | The USA | The prescriptive authority for nurse practitioners | Impendent nurse prescribing | FORC |
| Creedon, O’Connell [55] | 2009 | Ireland | The introduction of nurse prescribing to the Irish setting. | Independent and supplementary nurse prescribing | FORC, EDUC, PROR |
| Culley [56] | 2005 | The United Kingdom | Current prescribing options for nurses | Independent and supplementary nurse prescribing | FORC, LEGL, EDUC |
| Daly [57] | 2006 | The United Kingdom | The policies that led to developments in non-medical prescribing and implications for practice. | Independent and supplementary nurse prescribing | LEGL, EDUC |
| David, Brown [127] | 1995 | Sweden, the UK | The Swedish nurse prescribing system | Independent nurse prescribing | FORC, LEGL, EDUC |
| Davis, Drennan [128] | 2007 | The UK, the USA, New Zealand, Australia | The prescribing behaviours of community-based nurses and general practitioners. | Independent and supplementary nurse prescribing | FORC, LEGL, EDUC, PROR |
| Donato [58] | 2009 | The Netherlands | Nurse practitioners in the Netherlands. | Supplementary nurse prescribing | FORC, LEGL |
| Dragon [129] | 2008 | Australia, New Zealand | The prescribing patterns of advanced practitioners and why many are not working to capacity. | Independent nurse prescribing | LEGL, PROR |
| Durand [130] | 1998 | The UK, the USA | The restriction on role development of the ENP | Independent nurse prescribing | FORC |
| Elsom, Happell, Manias [131] | 2009 | Australia, the USA | The standard of care provided by nurse practitioners and medical practitioners. | Independent nurse prescribing | FORC, LEGL, EDUC |
| Evans [132] | 2009 | The UK and the USA | The historical context of mental health nursing and its relationship to nurse prescribing. | Independent and supplementary nurse prescribing | FORC, LEGL, EDUC |
| Faucher [59] | 1992 | The USA | The legal avenues used by nurse practitioners to obtain prescriptive authority | Independent nurse prescribing | FORC |
| Fisher [60] | 2005 | The United Kingdom | The impact of nurse prescribing on the relationships between prescribers, nurses, doctors, pharmacists, patients and carers. | Independent nurse prescribing | FORC, LEGL, EDUC |
| Forchuk, Kohr [61] | 2009 | Canada | The role of prescriptive authority for nurses within Canada | Independent and supplementary nurse prescribing, patient group directions | FORC, LEGL |
| Ford, Otway [62] | 2008 | The United Kingdom | The need for continuing professional development in the area of prescribing | Independent and supplementary nurse prescribing | FORC |
| Gallagher [63] | 2006 | The United Kingdom | The anticipated benefits of prescribing changes for patients receiving treatment for substance misuse. | Independent and supplementary nurse prescribing | LEGL, EDUC |
| Gilmour, Bickford [64] | 2007 | The United Kingdom | The development of patient group directions and independent and supplementary prescribing. | Independent and supplementary nurse prescribing, patient group directions | FORC, LEGL, EDUC, PROR |
| Goswell, Siefers [65] | 2009 | The United Kingdom | The experiences of ward-based nurse prescribers regarding the use of non-medical prescribing with the open formulary | Independent and supplementary nurse prescribing | FORC, LEGL, EDUC, PROR |
| Grassby [66] | 2005 | The United Kingdom | The prescription of controlled drugs. | Independent and supplementary nurse prescribing | FORC, LEGL, EDUC |
| Gray, Parr, Brimblecombe [67] | 2005 | The United Kingdom | The current activities and attitudes regarding supplementary nurse prescribing in psychiatric settings. | Supplementary nurse prescribing | FORC, EDUC |
| Green, Westwood, Smith et al. [68] | 2009 | The United Kingdom | The provision of continued professional development for non-medical prescribers. | Independent and supplementary nurse prescribing | FORC, LEGL |
| Greveson [69] | 2009 | The United Kingdom | The safety and efficacy of nurse independent prescribing in inflammatory bowel disease. | Independent and supplementary nurse prescribing | FORC, LEGL, EDUC, PROR |
| Griffith [70] | 2006 | The United Kingdom | Extension of the right to prescribe controlled drugs to independent, supplementary nurse prescribers | Independent and supplementary nurse prescribing, patient group directions | LEGL |
| Griffith [71] | 2007 | The United Kingdom | The legal requirements for the prescribing and administration of medicines. | Independent and supplementary nurse prescribing, patient group directions | LEGL, EDUC, PROR |
| Haidar [133] | 2007 | The UK and the USA | The skills of prescribing and clinically assessing patients. | Independent nurse prescribing | FORC, LEGL |
| Hall [72] | 2005 | The United Kingdom | Supplementary prescribing for nurses. | Independent and supplementary nurse prescribing, patient group directions | LEGL, EDUC, PROR |
| Hall, Cantrill, Noyce [73] | 2006 | The United Kingdom | The barriers that could either prevent community nurses from prescribing or reduce the number of times a nurse might prescribe. | Independent and supplementary nurse prescribing | FORC, LEGL |
| Hansen-Turton, Ritter, Valdez [74] | 2009 | The USA | Successful advocating of nurses for legislative reforms in Pennsylvania | Independent nurse prescribing | FORC |
| Harkless [75] | 1989 | The USA | The historical development of medical prescriptive authority | Independent nurse prescribing | FORC |
| Hemingway, McAllister, Bailey et al. [134] | 2006 | The UK and the USA | The clinical practice and educational preparation for prescriptive authority for nurses in US centres. | Independent nurse prescribing | FORC, LEGL, EDUC |
| Hemingway, Ely [76] | 2009 | The United Kingdom | Historical overview of- and the growth of mental health nurse prescribing in the UK. | Independent and supplementary nurse prescribing | FORC, LEGL, EDUC |
| Hinchliffe [77] | 2006 | The United Kingdom | The ‘All Wales’ Supplementary Prescribing training course | Independent and supplementary nurse prescribing | FORC, EDUC, PROR |
| Hobden [78] | 2007 | The United Kingdom | The legal options available for nurses to supply and administer medicines. | Independent and supplementary nurse prescribing, patient group directions | EDUC, LEGL |
| Houweling, Kleefstra, van Hateren et al. [119] | 2009 | The Netherlands | The transfer of management of type 2 diabetes from an internist to a nurse specialised in diabetes | Supplementary nurse prescribing | LEGL |
| Hughes, Lockyer [79] | 2004 | New Zealand | The introduction of nurse prescribing in New Zealand | Independent nurse prescribing | FORC, EDUC, PROR |
| Jacobs, Boddy [80] | 2008 | New Zealand | The conditions and forces in play in the development of advanced nursing practice in New Zealand. | Independent nurse prescribing | FORC |
| Jones [85] | 1999 | The United Kingdom | The implementation and future of nurse prescribing | Independent and supplementary nurse prescribing, patient group directions | FORC, LEGL, EDUC, PROR |
| Jones, Jones [135] | 2005 | The UK and the USA | Supplementary nurse prescribing and it’s potential application in a number of mental health settings. | Independent and supplementary nurse prescribing, patient group directions | FORC, LEGL,EDUC, PROR |
| Jones [81] | 2006 | The United Kingdom | Perceptions of nurses and psychiatrists concerning supplementary prescribing on acute psychiatric wards. | Independent and supplementary nurse prescribing, patient group directions | FORC |
| Jones [82] | 2006 | The United Kingdom | Impact of supplementary prescribing on relationships between nurses and psychiatrists | Independent and supplementary nurse prescribing | FORC, LEGL, EDUC |
| Jones [83] | 2008 | The United Kingdom | The implementation of independent  nurse prescribing in mental health settings | Independent and supplementary nurse prescribing | FORC, LEGL |
| Jones [84] | 2009 | The United Kingdom | The development of a prescribing role for acute care nurses. | Independent and supplementary nurse prescribing | FORC, LEGL, EDUC |
| Koch, Pazaki, Campbell [86] | 1992 | The USA | The first 20 years of nurse practitioner literature | Independent nurse prescribing | FORC |
| Latter, Mabel, Myall et al. [137] | 2007 | The UK and the USA | The prescribing competencies and standards of independent nurse prescribers. | Independent and supplementary nurse prescribing | FORC, LEGL, EDUC, PROR |
| Latter, Mabel, Myall et al. [136] | 2007 | The United Kingdom and Sweden | Independent nurse prescribers’ education and continuing professional development. | Independent and supplementary nurse prescribing | FORC, LEGL, EDUC |
| Latter [87] | 2008 | The United Kingdom | The safety and quality of independent nurse prescribers’ current practice. | Independent nurse prescribing | FORC |
| Lee, Fitzgerald [88] | 2008 | Australia | The development of an internship model for nurse practitioners. | Independent nurse prescribing | FORC, EDUC |
| Lilley, Marshall, McIntosh et al. [89] | 2005 | The United Kingdom | The introduction of nurse prescribing in a paediatric hospital setting. | Independent and supplementary nurse prescribing | FORC, LEGL, EDUC, PROR |
| Lim, Honey, Kilpatrick [138] | 2007 | New Zealand, the UK and the USA | The educational framework for teaching pharmacology to prepare  nurses for prescribing in New Zealand. | Independent nurse prescribing | FORC, LEGL, EDUC |
| Lockwood, Fealy [139] | 2008 | Ireland and New Zealand | The attitudes and perceived barriers of Irish clinical nurse specialists to nurse prescribing. | Independent and supplementary nurse prescribing | EDUC, LEGL |
| Lymn, Bath-Hextall, Wharrad [140] | 2008 | The UK, the USA and Australia | Evaluation of pharmacology education for nurse prescribing students. | Independent and supplementary nurse prescribing | FORC, EDUC |
| Manchester [90] | 1998 | New Zealand | The extension of prescribing rights to nurses | Independent nurse prescribing | FORC, EDUC |
| Meadows, Sheehan [91] | 2005 | The United Kingdom | The expanding role of the  rheumatology nurse. | Independent and supplementary nurse prescribing, patient group directions | LEGL, EDUC, PROR |
| O’Hare [92] | 2007 | The United Kingdom | The development of nurse prescribing within the cancer nursing team of a general hospital. | Independent nurse prescribing | FORC, LEGL, EDUC, PROR |
| Padmore [93] | 2005 | The United Kingdom | Nurse prescribing in diabetes care. | Independent and supplementary nurse prescribing, patient group directions | FORC, EDUC |
| Patel, Robson, Rance et al. [94] | 2009 | The United Kingdom | The attitudes of psychiatrists and nurses regarding mental health nurse prescribing. | Independent and supplementary nurse prescribing | FORC, LEGL, EDUC |
| Peet, van der [117] | 2010 | The Netherlands | The experimental law concerning the independent authority of the Nurse Specialist in the Netherlands | Independent prescribing | FORC, LEGL, EDUC |
| Peet, van der [118] | 2010 | The Netherlands | The law concerning the prescriptive authority of nurses in the Netherlands | Supplementary prescribing | FORC, LEGL, EDUC |
| Peniston-Bird [95] | 2007 | The United Kingdom | Consultation on non-medical prescribing. | Independent and supplementary nurse prescribing | LEGL, EDUC, PROR |
| Plonczynski, Oldenburg, Buck [96] | 2003 | The USA | The evolution of prescriptive authority for nurses in the United States | Independent nurse prescribing | FORC |
| Pollock, Dudgeon [97] | 2006 | The United Kingdom | The blockages, facilitating factors and current practice of nurse prescribing in Scotland. | Independent and supplementary nurse prescribing | FORC, LEGL, EDUC |
| Pontin, Jones [98] | 2007 | The United Kingdom | The opportunities  and barriers to nurse prescribing for children’s nurses, and the development of a training strategy. | Independent and supplementary nurse prescribing, patient group directions | FORC, LEGL |
| Ring [99] | 2006 | The United Kingdom | The challenges influencing the development, implementation and effectiveness of nurse prescribing. | Independent and supplementary nurse prescribing | EDUC, PROR |
| Ross [100] | 2009 | The United Kingdom | The barriers to implementing mental health nurse independent prescribing . | Independent and supplementary nurse prescribing | FORC, LEGL, EDUC |
| Ryan [102] | 2007 | The United Kingdom | Challenges and opportunities of nurse prescribing in child and adolescent mental health services. | Independent and supplementary nurse prescribing | FORC, LEGL |
| Ryan-Woolley, McHugh, Luker [101] | 2008 | The United Kingdom | The prescribing practice of and views of specialist nurses working in cancer and palliative care on nurse prescribing training. | Independent and supplementary nurse prescribing | LEGL, EDUC |
| Sheer, Wong [141] | 2008 | The UK, Canada, the USA, Australia and New Zealand | To examine the development of advanced nursing practice globally. | Independent nurse prescribing and patient group directions | LEGL, EDUC |
| Shuttleworth [103] | 2005 | The United Kingdom | The history of nurse prescribing in the UK and the most recent extension | Independent and supplementary nurse prescribing | FORC, LEGL |
| Skingsley, Bradley, Nolan [104] | 2006 | The United Kingdom | The development and content of a ‘top-up’ neuropharmacology  module for mental health nurses. | Independent and supplementary nurse prescribing | FORC, LEGL, EDUC |
| Snowden [105] | 2006 | The United Kingdom | Suggestion of a CMP for community psychiatric nurses prescribing for elderly with mental health needs. | Independent and supplementary nurse prescribing, patient group directions | LEGL, EDUC |
| Snowden [106] | 2006 | The United Kingdom | The impact of mental health nurse prescribing | Independent and supplementary nurse prescribing | FORC |
| Snowden [142] | 2008 | The UK and the USA | The history of the regulation of medicines. | Independent and supplementary nurse prescribing | FORC, LEGL, EDUC, PROR |
| Spence, Anderson [107] | 2007 | New Zealand | The implementation of a prescribing practicum within a Master's degree in advanced nursing practice. | Independent nurse prescribing | FORC, EDUC |
| Stenner, Courtenay [108] | 2008 | The United Kingdom | Nurse prescribers’ views on the role of inter-professional relationships and support for nurse prescribing in acute and chronic pain. | Independent and supplementary nurse prescribing | FORC, LEGL, EDUC |
| Stenner, Carey, Courtenay [109] | 2009 | The United Kingdom | Doctor and non-prescribing  nurse views about nurse prescribing in dermatology. | Independent and supplementary nurse prescribing | FORC, LEGL, EDUC |
| Strickland-Hodge [110] | 2008 | The United Kingdom | The expressed beliefs about the pharmacological knowledge of nurses in prescribing practice. | Independent and supplementary nurse prescribing | FORC, LEGL, EDUC |
| Tarmina [111] | 1982 | The USA | The prescriptive privileges of nurse practitioners in Utah | Independent nurse prescribing | FORC |
| Ulfvarson, Mejyr, Bergman [112] | 2007 | Sweden | The analysis of adverse drug reaction reporting by nurses. | Independent nurse prescribing | FORC, LEGL, EDUC |
| Warburton, Kahn [113] | 2007 | The United Kingdom | The standards for- and numeracy skills \of nurse prescribers. | Independent and supplementary nurse prescribing | LEGL, EDUC |
| Warner [114] | 2005 | The United Kingdom | The theory that underpins nurse prescribing | Independent and supplementary nurse prescribing | FORC, LEGL, EDUC |
| Wells, Bergin, Gooney et al. [115] | 2009 | Ireland | The views of community mental health nurses on nurse prescribing. | Independent and supplementary nurse prescribing | FORC |
| Wilkinson [116] | 2005 | The United Kingdom | Supplementary nurse prescribing  for overactive bladder. | Independent and supplementary nurse prescribing | FORC, LEGL, EDUC, PROR |

1. Dimensions of nurse prescribing being discussed:

   internal and external forces related to the introduction of legal nurse prescribing (FORC);

   legal conditions under which nurse prescribing of medicines will be or has been realized (LEGL);

   educational conditions under which legal nurse prescribing of medicines will be or has been realized (EDUC);

   practical-organizational conditions under which legal nurse prescribing of medicines will be or has been realized (PROR) [↑](#endnote-ref-2)
