# Supplementary material for: Nurse prescribing of medicines in Western European and Anglo-Saxon countries: a systematic review of the literature
Source: BMC Health Serv Res. 2011 May 27;11:127. doi: 10.1186/1472-6963-11-127 (PMC3141384; doi:10.1186/1472-6963-11-127)
Supplement: Additional file 3 — Results verification search with relevant stakeholders in Western European and Anglo-Saxon countries. [file 1472-6963-11-127-S3.DOC]

### Additional file 3 – Results of verification literature search with relevant stakeholders in Western European and Anglo-Saxon countries

| ***Country*** | ***Stakeholders*** | ***Results*** |
| --- | --- | --- |
| Australia | Australian Nursing Federation, Western Australian Department of Health | Confirmation received that nurses are allowed to prescribe medicines in all States and Territories in Australia. |
| Austria | Österreichischen Gesundheits- und Krankenpflegeverband, Federal Ministry of Health | Confirmation received that nurses are not allowed to prescribe medicines and no implementation process is being initiated. |
| Belgium | Association Belge des Syndicats Médicaux, FPS Public health- General Directory Healthcare Facilities Organization | Confirmation received that nurses are not allowed to prescribe medicines and no implementation process is being initiated. |
| Canada | Canadian Nurses Association, Canadian Medical Association | Confirmation received that nurses are allowed to prescribe medicines in all Canadian jurisdictions. |
| Denmark | Danish Nurses' Organization, Danish Medical Association, National Board of Health | Confirmation received that nurses are not allowed to prescribe medicines and no implementation process is being initiated, although the Danish Nurses’ Organization is currently lobbying to gain limited prescription rights for relevant groups of nurses. |
| Finland | Finnish Nurses Association | In contradiction to our literature findings, we were informed that an implementation process of legal nurse prescribing is currently being rolled out in Finland to allow a specific category of nurses the right to prescribe a limited number of medicines. |
| France | IRDES- Institute for Research and Information in Health Economics | Confirmation received that nurses are not allowed to prescribe medicines and no implementation process is being initiated. |
| Germany | German Nurses Association, Bundesärztekammer, Bundesministerium für Gesundheit | Confirmation received that nurses are not allowed to prescribe medicines and no implementation process is being initiated. |
| Iceland | Icelandic Nurses Association, Ministry of Health | Confirmation received that nurses are not allowed to prescribe medicines and no implementation process is being initiated. |
| Ireland | Irish Nurses and Midwifes Organisation, Irish Medical Organisation, Department of Health and Children | Confirmation received that nurses are allowed to prescribe medicines within Ireland. |
| Italy | Ministero della Sanita | Confirmation received that nurses are not allowed to prescribe medicines and no implementation process is being initiated. |
| Luxembourg | Luxemburg Nurses Association, Ministry of Health | Confirmation received that nurses are not allowed to prescribe medicines and no implementation process is being initiated. |
| Netherlands | Verpleegkundigen & Verzorgenden Nederland, Koninklijke Nederlandsche Maatschappij tot bevordering der Geneeskunst | Confirmation received that legal nurse prescribing is being implemented. |
| New Zealand | New Zealand Nurses Organisation, New Zealand Medical Association, Ministry of Health | Confirmation received that nurses are allowed to prescribe medicines within New Zealand. |
| Norway | Norwegian Nurses Organisation, Ministry of Health and Care Services | We were informed that Public Health Nurses for Children and Young Adults are allowed to prescribe the birth control pill for girls and young women (16 to 19 years old), for which they must complete a certified course. Moreover, over the last year there has been discussion about the establishment of a Nurse Practitioner Master program with possible inclusion of prescribing of medicines. |
| Portugal | Nursing Board of the Ordem dos Enfermeiros, Portuguese Medical Association | Confirmation received that nurses are not allowed to prescribe medicines and no implementation process is being initiated. |
| Spain | Universidad de Barcelona | Confirmation received that nurse prescribing is in a regulatory process in Spain. |
| Sweden | Swedish Nurses Association, Swedish Medical Association, Socialdepartementet | Confirmation received that certain nurses are allowed to prescribe medicines within Sweden. |
| Switzerland | Swiss Nursing Association (SBK-ASI), FMH Verbindung der Schweizer Ärztinnen und Ärzte | Confirmation received that nurses are not allowed to prescribe medicines and no implementation process is being initiated. |
| United Kingdom | The Royal College of Nursing, Department of Health | Confirmation received that nurses are allowed to prescribe medicines within the United Kingdom. |
| United States of America | American Nurses Association | Confirmation received that nurses are allowed to prescribe medicines within the United States of America. |
