# Supplementary material for: Nurse prescribing of medicines in Western European and Anglo-Saxon countries: a systematic review of the literature
Source: BMC Health Serv Res. 2011 May 27;11:127. doi: 10.1186/1472-6963-11-127 (PMC3141384; doi:10.1186/1472-6963-11-127)
Supplement: Additional file 4 — Description of nurse prescribing in nine Western European and Anglo-Saxon countries according to core themes. [file 1472-6963-11-127-S4.DOC]

**Additional file 4 – Description of nurse prescribing in nine Western European and Anglo-Saxon countries according to core themes**

***Australia***

| **Description of nurse prescribing in Australia** |
| --- |
| *Year of introduction:*  In Australia, the first group of nurses started prescribing in the year 2000 [5,129]. |
| *Forces which led to introduction of nurse prescribing:*  Predominantly because of a shortage of doctors in rural areas. Nurse prescribers had to meet the medication needs of patients in these remote areas [5,120,124,125,131]. Followed by a desire to develop the nurse practitioner role [88,131]. |
| *Legal conditions:*   - Regulated at: State/territory level [5,6,129,131] - Prescriptive authority:   - Independent prescriptive authority: Some nurse practitioners have independent prescriptive authority [6].   - In other states, nurses prescribe under standing directives put in place by a doctor [6,128]. - Protocols/formularies in place: In the state South Australia, every nurse practitioner has their own individual formulary of medicines from which to prescribe [129]. Other Australian states have general limited formularies for nurse prescribers in place [6,131], and a number of Australian states such as New South Wales and Queensland use protocols to facilitate nurses’ prescriptions [129]. - To whom can nurses prescribe: **-** - Formal responsibilities: Nurses must take full responsibility for patient’s treatment [5]. |
| *Educational conditions:*   - Place within educational system: Prescribing training is part of general Nurse Practitioner curricula[5,6,88,131]. - Level of prescribing training: Master level [5,6,88,131] - Admission criteria: In the state Victoria admission criteria to Victoria’s NP programme are a Bachelor of Nursing degree, at least 2 years of professional nursing experience, demonstration of advanced clinical practice, research, and leadership, usually requiring many years of experience and additional postgraduate qualifications in a particular area of nursing speciality [131]. - Content prescribing training: Pharmacological and therapeutical treatment elements, legal and ethical considerations of prescribing, focus on taking full responsibility for patient’s treatment [5]. |
| *Practical-organizational conditions:*   - Registration: - - Continuing professional development: - - Financial issues: - |

***Canada***

| **Description of nurse prescribing in Canada** |
| --- |
| *Year of introduction:*  In Canada, the first group of nurses started prescribing in the early 1990s [6]. |
| *Forces related to introduction:*  Nurse prescribing was introduced to meet the health service needs of patients in remote and isolated areas and to address the general shortage of doctors, particularly in remote areas. In addition, illegal prescribing by community health nurses could be formally recognized [6,61,124]. |
| *Legal conditions:*   - Regulated at: Provincial/territorial level [5,6] - Prescriptive authority:   - Independent prescriptive authority: In some states, nurses have independent prescriptive authority [5,6,61].   - In other states, medical directions offer the opportunity to develop protocols that allow nurses to prescribe medicines [5,61]. - Protocols/formularies in place: A number Canadian provinces use protocols to facilitate nurses’ prescriptions, others use formularies [5,6,61]. - To whom can nurses prescribe: In the province Ontario nurses can only prescribe in primary care, long-term care and outpatient clinics [61]. - Formal responsibilities: In the province British Columbia registered nurses who initiate medicines are ‘fully responsible and accountable’ for their prescription [61]. |
| *Educational conditions:*   - Place within educational system: Part of general Nurse Practitioner education [5,6,141] - Level of prescribing training: Most educational programmes for nurse practitioners are at postgraduate level [5,141] - Admission criteria: - - Content prescribing training: - |
| *Practical-organizational conditions:*   - Registration: - - Continuing professional development: - - Financial issues: - |

***Ireland***

| **Description of nurse prescribing in Ireland** |
| --- |
| *Year of introduction:*  In Ireland, the first group of nurses started prescribing in the year 2007 [5,55,115,139]. |
| *Forces which led to introduction of nurse prescribing:*  The striving for improvements in service delivery, integrated care, more cost-effectiveness, and to deploy the education and expertise of nurses more efficiently. This in the light of an ageing population [5,55,115]. |
| *Legal conditions:*   - Regulated at: National level - Prescriptive authority: Independent [5,139] - Protocols/formularies in place: Nurse prescribers may independently prescribe from an open formulary specific to their field of clinical practice [5,139]. - To whom can nurses prescribe: - - Formal responsibilities: - |
| *Educational conditions:*   - Place within educational system: Prescribing courses are offered on a stand-alone basis, i.e. they are not part of some regular nursing curriculum [5,55,139]. - Level of prescribing training: Level 8 in the Irish education system, comparable to Honours Bachelor Degree level [5]. - Admission criteria: Applicants must have a minimum of three years clinical experience post-registration, be competent to study at graduate level and appear on the live register of An Bord Altranais [5]. - Content prescribing training: The three core modules are: Professional accountability in nurse and midwife prescribing, drug action and therapeutics, and systematic assessment and evaluation in patient care. There is also a practicum component [5]. |
| *Practical-organizational conditions:*   - Registration: Nurse prescribers must be registered with the national nursing board An Bord Altranais [5,115,139]. - Continuing professional development: The responsibility for continuing professional development lies with each health service provider and the individual nurse prescriber and is focused on maintaining competence and promoting evidence based learning [5]. - Financial issues: - |

***Netherlands***

| **Description of nurse prescribing in the Netherlands** |
| --- |
| *Year of introduction:*  In the Netherlands, the first group of nurses is expected to start prescribing in the near future [5,117,118]. |
| *Forces which led to introduction of nurse prescribing:*  The striving for a more cost-effective healthcare system, the ageing of the population and a serious shortage of specialists and inner-city physicians led to the (further) development of the nurse practitioner role [58]. Developments in this role and the role of specialist nurses prompted the nurse prescribing initiative in the Netherlands [5]. Moreover, it is known that nurses in practice are already prescribing medicines for decades, which is illegal, and legalizing nurse prescribing can put a stop on this undesirable situation [118]. Furthermore, the government is striving for task reallocation in the health care sector [117,118]. |
| *Legal conditions:*   - Regulated at: National level - Prescriptive authority:   - Independent: for Nurse Specialists [117]   - Form resembling supplementary prescribing: for specific other categories of specialist nurses, most probably nurses specialized in caring for patients with diabetes, lung diseases and cancer [118]. - Protocols/formularies in place: Protocols will in the future be used to facilitate nurses’ prescriptions [58,119,118]. - To whom can nurses prescribe: - - Formal responsibilities: - |
| *Educational conditions:*   - Place within educational system: For Nurse Specialists, it is anticipated that prescribing will become an obligatory component of the Masters programme of Advanced Nursing Practice [5]. - Level of prescribing training: Nurse Specialists are prepared at Master level [5,117]. - Admission criteria: - - Content prescribing training: The course for Nurse Specialists has been developed based on the programme used for GPs in the Netherlands [5]. |
| *Practical-organizational conditions:*   - Registration: For Nurse Specialists, registering their title shows their prescriptive authority (during the experimental period of maximally 5 years) [117].  Prescriptive authority for specialist nurses will be linked to their possession of an educational certificate/qualification approved by the Minister of Health. Nurses can then request the Minister for a special endorsement in their BIG registration (registration system for health professionals kept up by the Ministry of Health) which shows their prescribing qualification [118]. - Continuing professional development: - - Financial issues: - |

***New Zealand***

| **Description of nurse prescribing in New Zealand** |
| --- |
| *Year of introduction:*  In New Zealand, the first group of nurses started prescribing in the year 2001 [5,6,122,138]. |
| *Forces which led to introduction of nurse prescribing:*  Nurse prescribing was introduced to meet the medication needs of patients in remote areas, improve patient care, increase the cost-effectiveness of the healthcare system, and make better use of the skills of the highly educated nursing workforce [5,6,22,79,80,90,107,120,122,125]. |
| *Legal conditions:*   - Regulated at: National level - Prescriptive authority: Independent [5,6]. - Protocols/formularies in place: New Zealand has general limited formularies for nurse prescribers in place [5,6,90,125]. - To whom can nurses prescribe: Prescriptive authority was for a long time only granted to nurses working in specific areas of care [90,125,138,139] but this recently appears to have expanded to include the whole NP scope of practice [5]. - Formal responsibilities: - |
| *Educational conditions:*   - Place within educational system: Preparation courses for nurse prescribing are offered within a Masters programme for advanced nursing practice or as a stand-alone Post Graduate Diploma (Prescribing) for nurses who already completed a Masters [5,6,22,27,40,49,107,138]. - Level of prescribing training: Master level [5,6,22,27,40,49,107,138]. - Admission criteria: Nurses must have at least 4 years of clinical experience in their speciality area [107,122]. - Content prescribing training: Core modules are: advanced health assessment, physiology and pathopohysiology, pharmacology, pharmodynamics, pharmokinetics, clinical decision-making skills and differential diagnosis. There is also a prescribing practicum. The mentor prescriber would also be a New Zealand practitioner in the same clinical area as the nurse [5,122,138]. |
| *Practical-organizational conditions:*   - Registration: Nurse prescribers must be registered with the New Zealand Nursing Council [5,6,79] - Continuing professional development: The New Zealand Nursing Council developed a comprehensive framework including requirements for ongoing competence. Nurses must provide evidence of the maintenance of their competencies in order to gain certification renewal [5,79]. - Financial issues: NPs have prescriber numbers so if a NP prescribes a drug the cost to the patient is the same as if a doctor prescribes [129]. |

***Spain***

| **Description of nurse prescribing in Spain** |
| --- |
| *Year of introduction:*  In Spain, the first group of nurses is expected to start prescribing somewhere in the future [5]). |
| *Forces which led to introduction of nurse prescribing:*  The Spanish General Council of Nursing was very active in the passing of the legislation to authorise nurses to “continue doing what they are already doing in the course of their daily practice”, i.e. to legalize the practices of nurses who were already prescribing for a long time [5]. |
| *Legal conditions:*   - Regulated at: The Medicine Law needs to be amended at national level to legally authorise nurse prescribing. However, health systems are being managed at a regional level by regional health ministers. The Regional Government of Andalusia has recently taken the initiative of leading the regulation of nurse prescribing [5]. - Prescriptive authority: The goal is to achieve the following four forms of prescriptive authority for nurses [5]:   - In accordance with institutional protocols and standardized health care plans.   - In accordance with protocols containing treatment based on personalized medical prescriptions (follow-up of chronic patients).   - In accordance with protocols relating to advanced nursing practice (specialities).   - Independent prescribing by means of a nurse dispensing order (prescription) to dispense all non-prescription medicines and health care products. - Protocols/formularies in place: - - To whom can nurses prescribe: - - Formal responsibilities: - |
| *Educational conditions:*   - Place within educational system: - - Level of prescribing training: - - Admission criteria: - - Content prescribing training: - |
| *Practical-organizational conditions:*   - Registration: - - Continuing professional development: - - Financial issues: - |

***Sweden***

| **Description of nurse prescribing in Sweden** |
| --- |
| *Year of introduction:*  In Sweden, the first group of nurses started prescribing in the year 1994 [5,6,85,112,125,127,136]. |
| *Forces which led to introduction of nurse prescribing:*  Nurse prescribing was introduced to offer patients, especially in remote areas, quicker and better access to medicines, reduce the workload of doctors, improve service to clients and make sure that primary care was given by an appropriate mix of health professionals [5,6,112,121,124,125,127]. |
| *Legal conditions:*   - Regulated at: National level - Prescriptive authority: Independent - Protocols/formularies in place: General limited formularies are in place for nurse prescribers [5,6,112,125-127]. - To whom can nurses prescribe: Only district nurses and nurses working in elderly care may prescribe for 60 conditions [5,6,85,125,127,136]. - Formal responsibilities: Nurses are not expected to make a decision about differential diagnosis before prescribing [127]. |
| *Educational conditions:*   - Place within educational system: Prescribing training is part of the Primary Health Care Specialist Nursing programme, undertaken by all district nurses [5]. - Level of prescribing training: The Primary Health Care Specialist Nursing programme is offered at Master level. - Admission criteria: Nurses must be under 60 [5,127]. - Content prescribing training: Pharmacology and drug treatment course [5]. |
| *Practical-organizational conditions:*   - Registration: - - Continuing professional development: - - Financial issues: - |

***United Kingdom***

| **Description of nurse prescribing in the United Kingdom** |
| --- |
| *Year of introduction:*  In the United Kingdom, the first group of nurses started prescribing nationally in the year 1998 [5,6,25,27,40,42,46,56,57,62,67-69,72,76,78,85,98,100,101,104,114,116,122,135] |
| *Forces which led to introduction of nurse prescribing:*  The effort to make better use of nurses’ skills and knowledge, and to improve the use of both professional’ and patients’ time. Provide a more streamlined, accessible and flexible health service, with more team working, and reduce waiting times  [21,25,26,29,31,32,34-37,39,42,46,47,51,52,62,64,67,69,76,82-84,87,89,92,93,100,102,103,106,109,116,124,125,130,132-134,137] |
| *Legal conditions:*   - Regulated at: National level - Prescriptive authority:   - Independent prescriptive authority (IP)   - Supplementary prescriptive authority (SP)   - Community practitioner prescriptive authority (CP)   - Use of Patient Group Directions (PGDs) - Protocols/formularies in place:   - IP: independent prescribers can prescribe from the entire British National Formulary (BNF), including unlicensed medicines and some controlled drugs [5,24,26,35,44-46,49,51-53,63,65,68,69,76,78,83,84,92,94,100-103,105,108,109,113,126,128,136,137,139].   - SP: Supplementary prescribers in the UK can prescribe from the entire BNF including all controlled drugs, provided they are listed in a clinical management plan agreed by the independent prescriber, nurse and patient [5,21,24,26,27,35,38,40,43-45,48-53,63,76,78,82,95,100,101,108,114,123-126].   - CP: Community practitioner nurse prescribers in the UK however, have their own more limited formulary to prescribe from [5,27]. - To whom can nurses prescribe: Community practitioner nurse prescribers can prescribe for a number of common conditions, but both independent- and supplementary nurse prescribers can prescribe for any medical condition or patient group within their clinical competence [5,25,27,35,40,50,52,63,68,69,71,78,84,89,92,94,95,100-102,105,109]. PGDs can in principle also be drawn up for any medical condition, but should be reserved for those situations where it offers ‘an advantage for the patient without compromising patient safety’ [40,72]. - Formal responsibilities:   - IP & CP: Independent nurse prescribers and qualified community nurse prescribers are responsible for the clinical assessment and diagnosis of a patient and for decisions about the clinical management required, including prescribing [27,40,47,48,51,56,69,72,78,83,84,89,92,100,102,108-110,114,124-126,136-138].   - SP: Supplementary prescribers are responsible for the continuing care of a patient, including prescribing, whilst the collaborating independent prescriber shares the responsibility for prescribing and holds full responsibility for the assessment and diagnosis of a patient [25,40,47-49,51,56,63,69,72,78,84,100,103,123,124,135]. |
| *Educational conditions:*   - Place within educational system:   - IP & SP: Independent- and supplementary prescribing courses are combined into a ‘dual qualification’ and offered on a stand-alone basis, i.e. they are not part of some regular nursing curriculum [5,32,33,44,51,53,64,72,93,110,116,122-126].   - CP: Training to prescribe from the British Nurse Prescribers Formulary for Community Practitioners is incorporated into Specialist Practitioner Programmes [5,6,22,27,40,49,107,140].   - PGDs: No specific training is required for nurses using PGDs, although most individual Trusts provide some in-house training [24,39,40,105]. - Level of prescribing training: IP & SP: prescribing courses are taught at undergraduate level 3 (degree level) [5,6,24,27,29,37,48,50,52,56,60,63-65,69,72,76,77,84,89,91,95,99,109,116,122-125,137,140,142]. - Admission criteria: Three years of clinical experience are required, of which the last year must be in the clinical field in which they intend to practice[5,29,42,53,71,84,108,109,124,126,137,140]. Another important requirement is nurses’ ability to demonstrate clinical assessment and clinical decision making skills [37,38,42,53,65,84,95,99,137]. Additional prerequisites for potential nurse prescribers include nurses’ ability to arrange a Designated Medical Practitioner (DMP) who will supervise them during their practice period and they must occupy a post in which nurse prescribing will enhance patient care [5,26,40,50,52,53,77,95,97,108,109,125]. - Content prescribing training: IP & SP: Consultation skills and decision making, influence on and psychology of prescribing, clinical pharmacology including the effects of co-morbidity, evidence based practice and clinical governance, calculation skills, promoting medicines concordance, legal, policy and ethical aspects, professional accountability and responsibility, prescribing in a team context, prescribing in the public health context, influence of pharmaceutical industry [48-50,52,110,114,122,124,125,132,136]. |
| *Practical-organizational conditions:*   - Registration: Nurse prescribers must have an annotation on the Nursing and Midwifery Council (NMC) register as a nurse prescriber [5,22,23,29,38,42,71,77,85,91,92,95,116,142]. - Continuing professional development: The responsibility nurses have in maintaining competence in prescribing falls into three areas: personal responsibility, employer responsibility and accessing CPD, the latter responsibility being a joint one between the nurse and the employer. CPD requirements are the same for independent, supplementary and community nurse prescribers. The NMC developed a guidance document and the National Prescribing Centre has produced a range of CD ROMs for nurse prescribers to support their continuing professional development (CPD). Nurses are responsible for remaining up-to-date with any changes in the prescribing initiative, including additions to their formularies [5,27,32,33,35,40,50,60,62,87,89,136,137]. - Financial issues: Funding to undertake nurse prescribing training is made available from central government through local level organizations, such as workforce development confederations, strategic health authorities and local NHS Trusts [40,42,46,47,65,69,72]. Access to a prescribing budget needs to be created for nurse prescribers before they can perform their role [27,85]. |

***United States of America***

| **Description of nurse prescribing in the United States of America** |
| --- |
| *Year of introduction:*  In the United States of America, the first group of nurses started prescribing in the 1960s. [5,6,30,107,115,123,128,134,135,138]. |
| *Forces which led to introduction of nurse prescribing:*  Nurse prescribing in the USA followed the development of the Advanced Practice Registered Nurse role [5,124]. The general ANP role, and hence prescriptive authority, was introduced to alleviate the shortages of medical practitioners in primary care and meet the medication- and healthcare needs of patients in remote areas [5,6,28,30,41,54,59,74,86,131,134,138]. Other incentives were the striving to increase the cost-effectiveness of the healthcare system [30,54,134], make better use of nurses skills [30,41,75,125,134] and legalise covert prescribing practices by nurses [75,96,111,130]. |
| *Legal conditions:*   - Regulated at: State level [5,6,28,85,125] - Prescriptive authority: In over half of the US states nurses have full independent prescriptive authority, whereas in other states mandatory collaboration with and/or supervision by a physician is required [5,6,28,54,59,75,96,124,135,137] - Protocols/formularies in place: A number of American states have limited formularies for nurse prescribers in place [5,6,28,85]. In other American states, for example Texas, nurses prescribe via protocols [28,133]. - To whom can nurses prescribe: - - Formal responsibilities: In the state Massachusetts nurses assume responsibility for prescribing [134]. |
| *Educational conditions:*   - Place within educational system: Training for nurse prescribing is linked to educational preparation for nurse practitioner roles [5,124,134]. - Level of prescribing training: In most states Master level [5,6,28,123,132,134,140,142]. - Admission criteria: - - Content prescribing training: - |
| *Practical-organizational conditions:*   - Registration: Nurse prescribers must register their qualification with their respective regulatory nursing bodies [134]. - Continuing professional development: - - Financial issues: In several states of the U.S.A., the social welfare program Medicaid does not reimburse prescriptions written by nurses [135]. |
